# Hapke-based computational method to enable unmixing of hyperspectral data of common salts

**DOI:** 10.1186/s13065-018-0460-z

**Published:** 2018-08-09

**Authors:** Fares M. Howari, Gheorge Acbas, Yousef Nazzal, Fatima AlAydaroos

**Affiliations:** 1 College of Natural and Health Sciences, Zayed University, P.O. Box 144534, Abu Dhabi, UAE; 2 UAE Space Agency, P.O. Box 7133, Abu Dhabi, UAE

**Keywords:** Reflectance spectroscopy, Halite, Gypsum, Reflectance parameters, Unmixing

## Abstract

Environmental scientists are currently assessing the ability of hyper-spectral remote sensing to detect, identify, and analyze natural components, including minerals, rocks, vegetation and soil. This paper discusses the use of a nonlinear reflectance model to distinguish multicomponent particulate mixtures. Analysis of the data presented in this paper shows that, although the identity of the components can often be found from diagnostic wavelengths of absorption bands, the quantitative abundance determination requires knowledge of the complex refractive indices and average particle scattering albedo, phase function and size. The present study developed a method for spectrally unmixing halite and gypsum combinations. Using the known refractive indexes of the components, and with the assistance of Hapke theory and Legendre polynomials, the authors develop a method to find the component particle sizes and mixing coefficients for blends of halite and gypsum. Material factors in the method include phase function parameters, bidirectional reflectance, imaginary index, grain sizes, and iterative polynomial fitting. The obtained Hapke parameters from the best-fit approach were comparable to those reported in the literature. After the optical constants (*n*, the so-called real index of refraction *and k*, the coefficient of the imaginary index of refraction) are derived, and the geometric parameters are determined, single-scattering albedo (or ω) can be calculated and spectral unmixing becomes possible.

**Electronic supplementary material:**

The online version of this article (10.1186/s13065-018-0460-z) contains supplementary material, which is available to authorized users.

## Introduction

Interest has grown in hyperspectral imaging and remote sensing for environmental analysis as it is inexpensive and fast and does not harm the environment in comparison to tradition soil analysis methods [1–3]. The hyper-spectral technique collects light absorbance and transmittance data from materials. The various earth materials differ from each other in their chemical and physical properties, leading to differences in their reflectance and absorption of light at different wavelengths. These differences are the basis for analyzing and classifying these material [4–7]. Experimental earth material models have been used to better understand their spectral signatures and to answer some related questions. Salt and evaporite minerals are common earth materials that can be investigated for their reflectance parameters [1, 4–6]. There is much interest in them since they have simple mineralogy yet significant environmental impacts on soils and plants. However, collected spectral data cannot be directly visually interpreted. Spectral pretreatment techniques, such as data normalization, continuum removal, etc., must be applied to smooth spectral graphs.

**Fig. 1:**
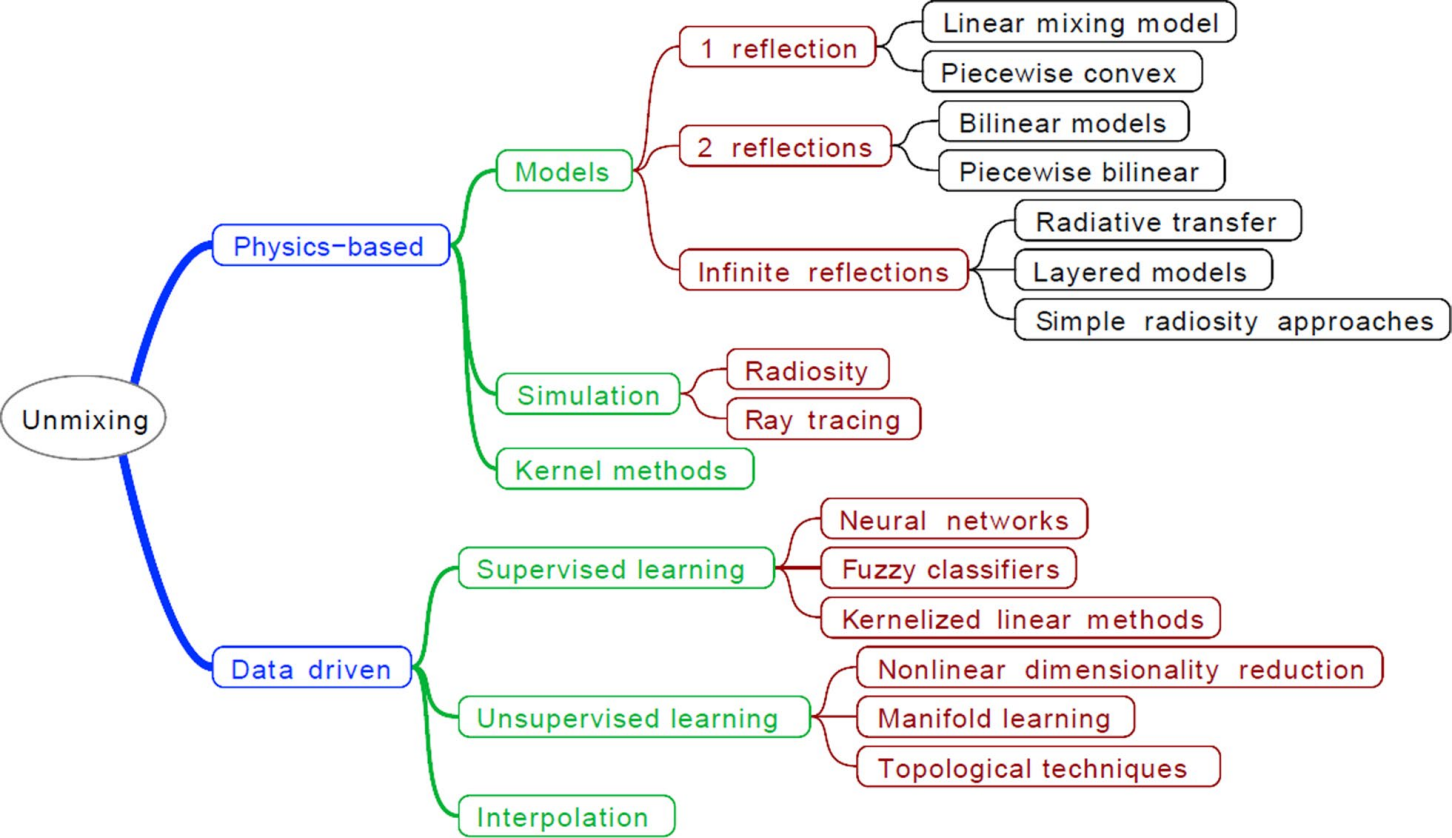
Taxonomic tree of the different unmixing techniques presented in literature [14]

One of the outstanding problems facing hyperspectral methods is the purity issue, i.e. how to relate the spectral properties of mixtures to the diagnostic characteristics of their components. Spectral unmixing is the procedure by which the spectrum of a mixed pixel is decomposed into a collection of constituent spectra or end members and a set of corresponding fractions or abundances of components. To solve this, two approaches are usually used (1) the semantic approach by tracing the diagnostic spectral features such as the location, shape and depth of the absorption bands for the pure components (endmembers), and relating these diagnostic features to the spectrum of the mixture of the components [3, 7–9]; and (2) the mathematical and statistical approach through equations or models that describe the reflectance process in terms of the variables that control light reflections [10–13]. Figure 1 shows the taxonomic tree of different unmixing techniques, which are presented and discussed in the literature [14]. The present study will briefly review linear mixing, with an emphasis on the Hapke model. For fractional mixtures, the linear mixing model is widely used. The linear mixing model assumes a well-defined proportional table of materials with a single reflection of the illuminating solar radiation. The observed spectrum ‘Y’ for any pixel can be expressed as:

1 $$\begin{aligned} {\text{Y}} &= ~{A_1}{S_1} + {A_2}{S_2} + \cdots + {A_x}{S_x} + {\text{W}} \\& = \mathop \sum \limits_{i = 1}^m {A_x}{S_x} + {\text{W}} \\ &= {\text{A}}_{\text{S}} + {\text{ W}} \end{aligned}$$

*A*_*i*_: fractional abundance of the *i*th endmember spectrum; *S*_*x*_: *x*th end member spectrum; Y: observed spectrum; W: error term for additive noise; S: matrix of end members.

If we have K spectral bands, and we denote the *x*th endmember spectrum as S_*x*_ and the abundance of the *i*th endmember as A_i_, the observed spectrum is Y for any pixel, accounting for additive noise (including sensor noise, endmember variability, and other model inadequacies). This model for pixel synthesis is the linear mixing model (LMM).

For example, consider deciduous reflectance (R_dec_) is 10% and spruce reflectance (R_spr_) is 50% and reflectance measured for the pixel (R_pix_) is 30. The mixing model for this example will be as:

2 $${\text{R}}_{\text{pix}} = \, \left( {{\text{A}}_{\text{dec}} *{\text{ R}}_{\text{dec}} } \right) \, + \, \left( {{\text{A}}_{\text{spr}} *{\text{ R}}_{\text{spr}} } \right)$$

Substitute values:

3 $$30 \, = \, \left( {{\text{A}}_{\text{dec}} *{ 1}0} \right) \, + \, \left( {{\text{A}}_{\text{spr}} *{ 5}0} \right)$$

Recognizing that all fractions must sum to 1 i.e. (A_dec_ + A_spr_) = 1; one can rearrange, substitute and solve via:

4 $${\text{A}}_{\text{spr}} \, = { 1 } - {\text{ A}}_{\text{dec}}$$

On the other hand, for intimate mixtures, the non-linear mixing approach has been tested and used [9]. The arrangement of components is not in an order because the components comprising the medium are not organized proportionally on the surface. The intimate mixture of materials results when each component is randomly distributed in a homogeneous way. Non-linear mixing is described by Hapke theory.

In Hapke theory, the isotropic multiple scattering approximation (IMSA) is often used to derive the diffuse reflectance of an intimate mixture, and combines two terms: the contribution of singly scattered light is given exactly, while the multiply scattered light is described by an approximate solution to the radiative transfer equation (RTE) for isotopically scattering particles [14]. One solves the RTE in an infinitely thick half-space of dispersed particulate matter. The derivation assumes that the particles are much larger than the wavelength of light, and uses geometrical optics arguments to solve the radiative transfer integral equations. IMSA considers large phase angles, B(g) = 0 and isotropic scattering, p(g). The objective of the present study is to use Hapke parameters from literature and fitting techniques to simulate and unmix spectra of a simple salt or evaporite system. The selected system is gypsum and halite and their mixtures. These salts have been selected because they are very commonly present in the soils of arid and semi-arid regions.

It was predicted that an intimate mixture of powders may be linearized in the single-scattering albedo [15]. For example, various mixtures of olivine, anorthite, enstatite and magnetite were studied [4]. This research [4] estimated the single-scattering albedo from bi-directional reflectance measurements, and converted the estimated mixing coefficients to mass fractions using the density of the endmembers. While other researchers demonstrated this technique for plagioclase-dominated minerals, computing the density from electron microprobe measurements [16]. Similarly, Hapke model was applied as a basis for unmixing of various mineral mixtures [17]. They replaced the measurement of density with further reflectance measurements. Other studies used the real and imaginary part of the optical constant to compute a quantitative abundance estimate [10]. This study provides a quantitative estimate of the abundance of halite and gypsum from spectral reflectance data, using Hapke model.

## Methodology

### Experimental design

In this study, laboratory experiments have been carried out under controlled conditions for the preparation of pure gypsum and halite crusts and their mixtures. Analytical grade compounds of NaCl (halite), and CaSO_4_·2H_2_O (gypsum) were used specifically. The weight fraction, grain size, type of mixing and mixing ratios are the main experimental variables.

### Data presentation

Different approaches of data processing were considered. The traditional method of graphing the spectral data was used. This method involves plotting the percent of reflectance against wavelength for the entire spectral region. Another method is the continuum removal, which is of significance in the study of the absorption features [9]. The continuum is the background absorption onto which the absorption features are superimposed. The continuum removal method implies the removal of the absorption features in the spectra, by plotting the intensities or band depths of the absorption features against the associated wavelengths. This technique of spectral reconstruction can isolate the spectral features and set them on a level, so that comparisons can be made [9].

### Unmixing model

The Hapke model describes the interaction of light with a medium, consisting of closely packed and randomly oriented particles (grains) [19]. In this model the bidirectional reflectance (the ratio of scattered irradiance to the source irradiance) is given below:

5 $$r\left( {\mu , \mu_{0} ,g} \right) = S\frac{\omega }{{4\pi \left( {\mu + \mu_{0} } \right)}}\left\{ {\left[ {1 + B\left( g \right)} \right]P\left( g \right) + H\left( \mu \right)H\left( {\mu_{0} } \right) - 1} \right\}$$

The variables μ and μ_0_ are the cosines of the reflection and incidence angles; g is the phase angle; B(g) is the back-scattering function, which defines the increase in brightness of a rough surface with decreasing phase; P(g) is the single-particle phase function; and the H(μ) is the isotropic scattering function. The main parameter is *ω*, the single scattering albedo, defined as the probability that the radiation would be scattered by the particle (power scattered to total power absorbed and scattered). The single scattering albedo can be expressed in term of optical constants n, k and the effective grain size 〈D〉 (the average distance traveled by rays that traverse the particle once, without being internally scattered); *ω* would thus be dependent on the wavelength of radiation (through n and k) and the shape and size of the particles (〈*D*〉 ≅ 0.9*D* for spherical particles, and departures from sphericity will decrease 〈*D*〉 further).

6 $$\omega = S_{e} + \left( {1 - S_{e} } \right)\frac{{1 - S_{i} }}{{1 - S_{i} {\Theta}}}{\Theta}$$

where *R(0)* is the surface reflection coefficient for externally incident light:

7 $$R(0) = \frac{{\left( {n - 1} \right)^{2} + k^{2} }}{{\left( {n + 1} \right)^{2} + k^{2} }}$$

Equation 7 is the specular reflection coefficient at normal incidence. An approximate expression for Se, valid if k is small, can be found by adding 0.05 to the specular reflection. If k is not small a more general expression for Se is given by

8 $${\text{Se }} = \, 0.0 5 8 7 { } + \, 0. 8 5 4 3\;R\left( 0 \right) \, + \, 0.0 8 70\;R\left( 0 \right)^{ 2}$$

*S*_*i*_ the reflection coefficient for internally scattered light is given by:

9 $$S_{i} = 1 - \frac{4}{{n\left( {n + 1} \right)^{2} }}$$

And Θ the transmission function of the grain is given by:

10 $${\Theta} = \frac{{r_{i} + \exp \left( { - \sqrt {\alpha \left( {\alpha + s} \right)\left\langle D \right\rangle } } \right)}}{{1 + r_{i} + \exp \left( { - \sqrt {\alpha \left( {\alpha + s} \right)\left\langle D \right\rangle } } \right)}}$$

11 $$\alpha = \frac{4k\pi }{\lambda }$$

12 $$r_{i} = \frac{{1 - \sqrt {\frac{\alpha }{\alpha + s}} }}{{1 + \sqrt {\frac{\alpha }{\alpha + s}} }}$$

Internal bi-hemispherical reflectance is *r*_*i*_ and *α* is internal absorption coefficient, while $\lambda$ is the wavelength of the photons.

*H* is Chandrasekhar integral multiple scattering function:

13 $$H\left( x \right) = \frac{1}{{1 - \omega x\left[ {r_{0} + \frac{{1 - 2r_{0} x}}{2}\ln \left( {\frac{1 + x}{x}} \right)} \right]}}$$

14 $$r_{0} = \frac{{1 - \sqrt {1 - \omega } }}{{1 + \sqrt {1 - \omega } }}$$

B is the backscattering function:

15 $$B\left( G \right) = \frac{{B_{0} }}{{1 + \frac{1}{h}\tan \left( {\frac{g}{1}} \right)}}$$

h (0 ≤ h ≤ 1) is the angular width and B_0_ (0 ≤ B0 ≤ 1) the amplitude of the opposition effect.

*P*(*g*) is the particle scattering phase function and describes the angular pattern into which the power is scattered. Where g = i − e is the phase angle. This function can be modeled by Legendre polynomials:

16 $$P\left( g \right) = 1 + b\cos \left( g \right) + c\left[ {1.5\cos^{2} \left( g \right) - 0.5} \right]{\text{ or }}P(g) = L_{0} (\cos (g)) + b*L_{1} (\cos (g)) + c*L_{2} (\cos (g)),\;{\text{as}}\; L_{0} = 1,\;L_{1} = x\quad{\text{and}} \quad L_{2} = 1/2(3x^{2} - 1),\;c < b$$

Or a double Henyey-Greenstein function:

17 $$P\left( g \right) = \left( {1 - c} \right)\frac{{1 - b^{2} }}{{\left( {1 + 2b\cos \left( g \right) + b^{2} } \right)^{{\frac{3}{2}}} }} + c\frac{{1 - b^{2} }}{{\left( {1 - 2b\cos \left( g \right) + b^{2} } \right)^{{\frac{3}{2}}} }}$$

where *b* (0 ≤ b ≤ 1) characterizes the anisotropy of the scattering lobe: b = 0 isotropic case, b = 1 single direction diffuser and c(0 ≤ c ≤ 1) backscattering fraction, characterizes the main direction of the diffusion, c < 0.5 representing forward scattering, and c > 0.5 representing backward scattering. In an intimate mixture of different minerals, bidirectional reflectance $r\left( {\mu , \mu_{0} ,g} \right)$ would depend nonlinearly on the abundances of each mineral component. On the other hand, the single-scattering albedo of a mixture of grains *ω*_*mix*_, is a linear combination of the single-scattering albedos of its individual endmembers, $\omega_{i}$:

18 $${\omega}_{mix} = \mathop \sum \limits_{i} f_{i} \omega_{i}$$

*f*_*i*_ is fractional relative cross section of component i:

19 $$f_{i} = \frac{{\sigma_{i} }}{{\mathop \sum \nolimits_{i} \sigma_{i} }}$$

20 $$\sigma_{i} = \frac{{m_{i} }}{{\rho_{i} D_{i} }}$$

*m*_*i*_ is mass abundance, *ρ*_*i*_ is density, *D*_*i*_ is the grain size of component i in the mixture. Thus, the reflectance spectra can be inverted to determine the mass abundance and grain sizes of the endmembers in the mixture. These equations and associated python code are provided in the Additional files 1, 2 and 3.

The Hapke model can be considered as an optimization problem through which we try to fit the data to a model that depends on a set of parameters. Since there are so many parameters it is practical to use optical and literature data to reduce the indeterminacies (over-fitting). Phase function parameters from measurements of the bi-directional reflectance at several phase angles are often used to determine some geometric parameters. To this end, one must measure the same reference sample in seven or more geometries, varying the incidence and emergent angles. This, however, is time consuming. For gypsum we used related results in Mustard and Pieters [4]. For halite the same geometrics values were assumed. Reducing the uncertainties in these values yields better fits and reduces the uncertainties in the statistical results, but does not significantly change the results for these samples. However, while optimizing the Hapke model, each grain size parameter usually requires separate measurements for gypsum [12] and for halite [18]. The method of Robertson et al. [10] was used, i.e. n was assumed to be known and the reflectance model was inverted to derive the effective grain size D and k. Also, with n values, Kramers–Kronig relations could be used to obtain the real and imaginary index of refraction from bidirectional reflectance measurements, though this requires larger spectra extending to UV and MIR. Since we had one reference sample with unknown grain sizes, D and k were kept free, but the starting k values were taken from the literature for gypsum. This provided starting values for effective grain size. Because of this approach, k values differ slightly from those in the literature, as the values also depend on other factors, e.g. hydration. For halite, k has not been sufficiently well studied in the literature. Halite is problematic, and is unique in that the single scattering albedo and the absorption values place it in a region where uncertainties are large. To determine the k values for halite the same procedure was used, again keeping the effective grain size as a free parameter. The difference is that the grain size of gypsum was taken as a starting parameter, assuming the two samples were prepared in the same way, to plot the results.

### Inversion algorithms

If the optical material parameters n and k, internal scattering s, the porosity S and the phase function parameters b and c are given, the reflectance spectra can be inverted to determine the mass abundance and grain sizes of the endmembers in the mixture. The phase function parameters b and c are determined by taking measurements of bidirectional reflectance at several angles, g [4]. Also, the wavelength-dependent real and imaginary indices of refraction can be obtained from bidirectional reflectance of samples with different grain sizes [13]. There are two general algorithms which were used to extract the mass abundances and the grain sizes of the endmembers in the mixture, from the model and measured reflectance.

The first approach [15, 19–21] is to find best fitting parameters *m*_*i*_, *D*_*i*_ that minimize the root mean square of the difference between the model and data reflectance. The second method is the probabilistic method [6], that uses a Markov Chain Monte Carlo algorithm and Bayes Theorem to estimate the probability density functions of the model parameters, given the reflectance data and model relationship between parameters. One of the advantages of the probabilistic model is that the detection noise model (which can be non-Gaussian for low count photons per pixel) can be accounted in the calculations.

While the first approach supplies a single set of data for the endmember mass fractions and particles sizes, the probabilistic model gives a range of values and, in principle, can account for non-unique solutions in the model parameters.

## Results and discussion

**Fig. 2:**
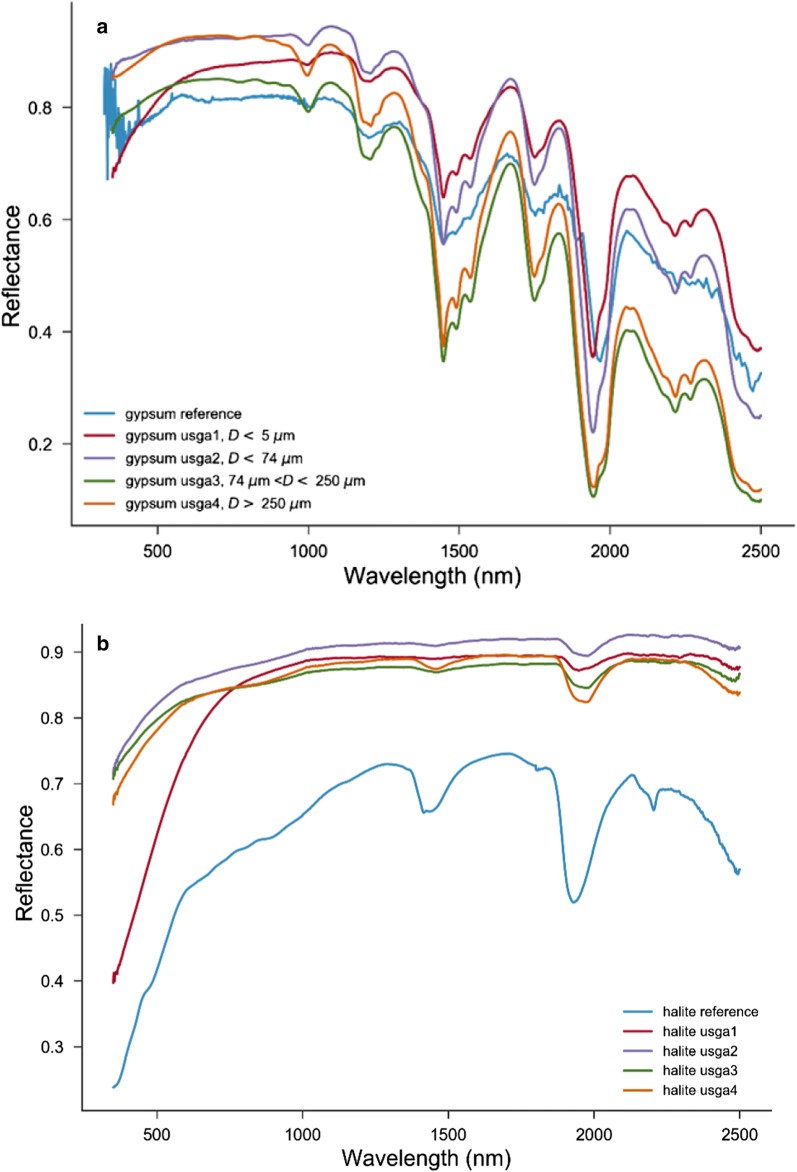
Spectra of the prepared gypsum (**a**) and halite (**b**) in comparison with the spectra from USGS

**Fig. 3:**
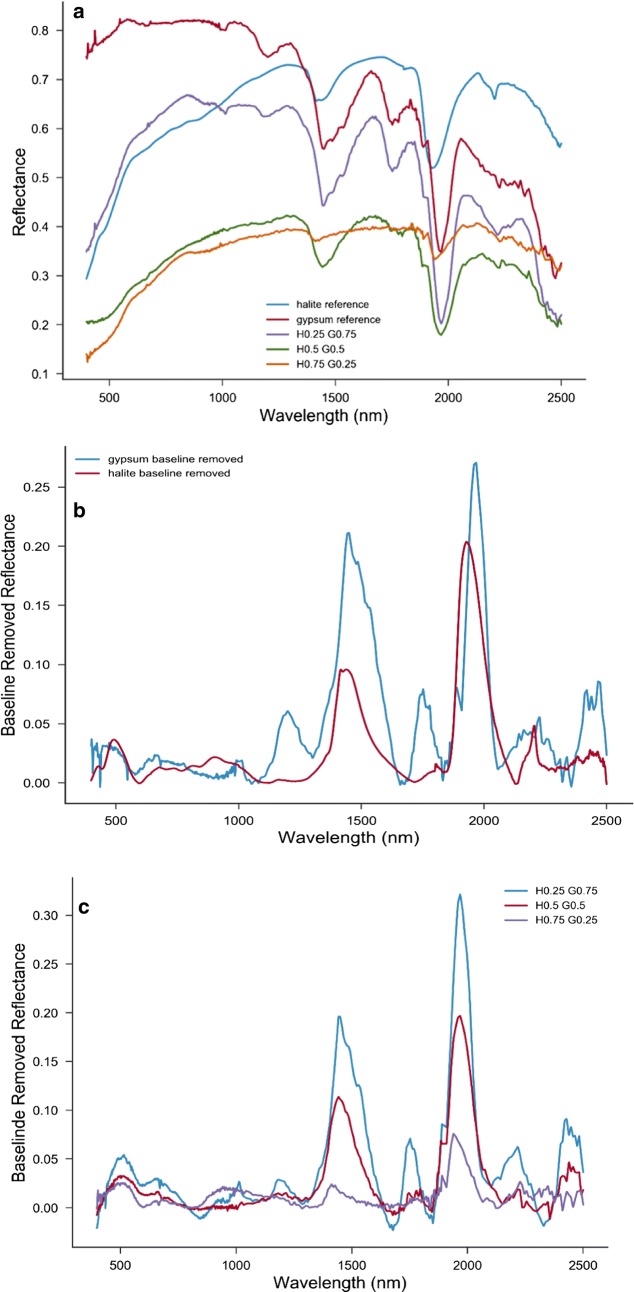
Spectra of gypsum and halite with their mixtures (**a**) in comparison of their continuum free spectra (**b**, **c**)

Figures 2 and 3 show the spectral profiles of halite and gypsum and their mixtures at different ratios. The profile shape for each endmember is unique and is easily distinguishable, one from the other. The differences in the shapes of the spectral profiles mainly result from differences in grain size and impurities. In the endmember spectra, gypsum has multiple absorption bands in the 300–2500 nm wavelength range, making it easily identifiable [8, 9]. The spectral frequencies are associated with the vibrational modes of the water molecules in the mineral structure. Similarly, the molecular vibration of water (O–H bonds vibrations) leads to absorption dips in the reflectance spectra of halite. However, there are noted differences between the two spectra making the distinction between the two minerals possible. To determine the band position, the study extracted the continuum spectra by iterative polynomial fitting of the reflectance data. This procedure helps to remove the shifts in the position of the bands due to the different slopes of continuum baseline spectra of the minerals. With the baseline removed, the spectra show that halite and gypsum have common bands at 1450 nm, 1950 nm and 2200 nm. However, for the gypsum the 1450, 1950 and 2200 nm bands consist of up to three overlapped bands. In addition, gypsum has distinct absorption bands at 950 nm, 1200 nm and 1750 nm.

**Fig. 4:**
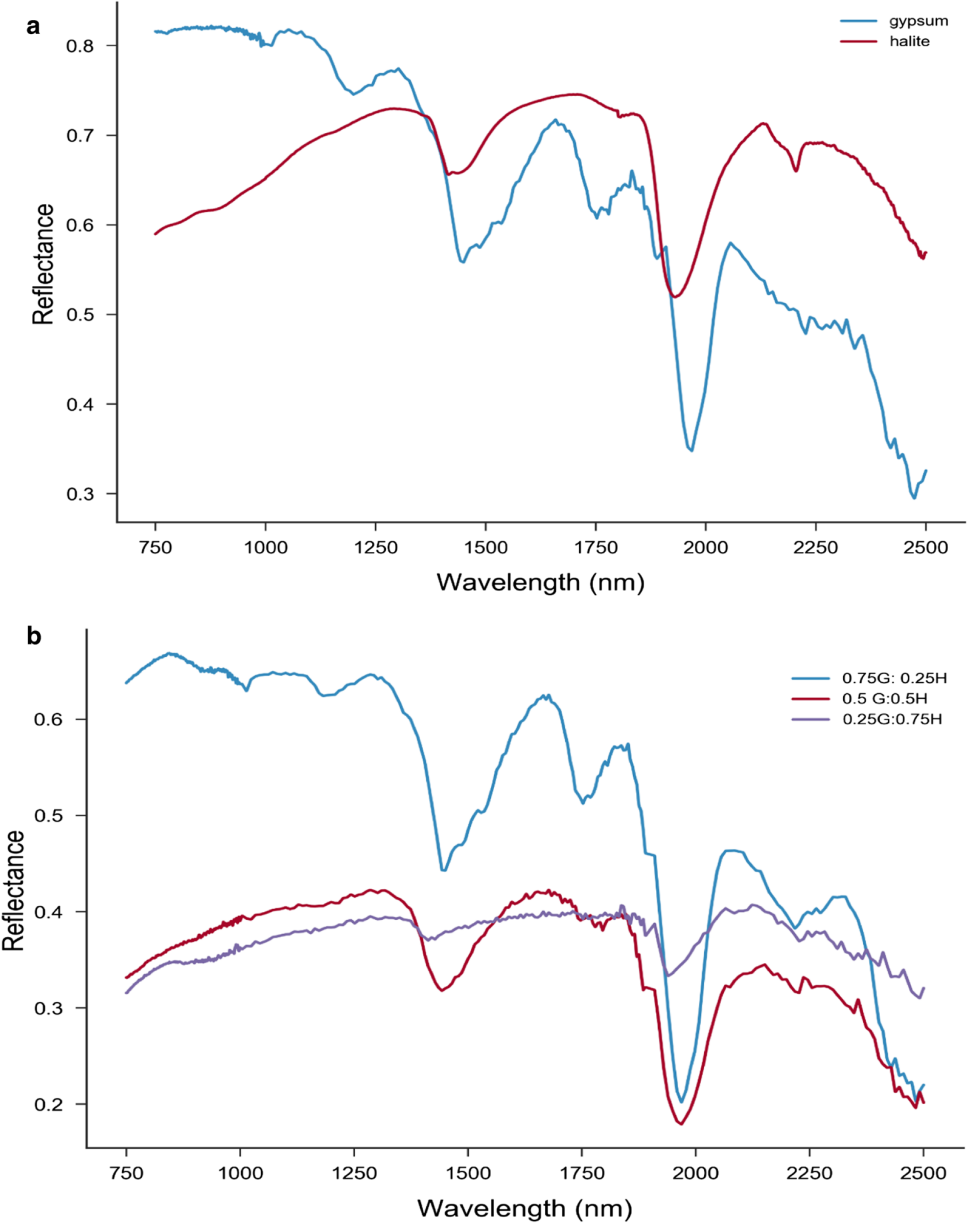
The spectral profile of gypsum and halite (**a**) and their mixtures (**b**) in the range of 750–2500 nm

The three distinct peaks make possible the detection of the presence of gypsum in the mixtures (Fig. 3), with the band depths depending on the gypsum concentration in the mixture. There is a notable sharp decrease in the 1750 and 2200 nm gypsum bands from 0.75 to 0.5 nominal. The present study deals only with the 750–2500 nm wavelength range, in accord with Robertson [10], as shown in Fig. 4, mainly because of the significant variation in the spectral profiles slope and the significant noise in the spectral region of 250–700 nm. Another reason is that halite has a strong absorption in UV, where n also varies strongly. The UV absorption band extends to VIS, making measurements and estimation of k values uncertain. Also, the single scattering albedo of gypsum is flat in the entire VIS range and ω values lie close to 1.

Polynomial fitting of the smooth background is a common algorithm used in peak fitting software. The idea is to keep the polynomial series low in degree (minimizing the number of parameters-Occam’s razor). Thus, the iterative algorithm is looking for that series that approximate the background satisfactory. This fitting is necessary to get a (qusi) quantitative understanding of the contributions of the components in the mixture to the reflection spectra, assuming linear mixture model is valid, from the size of the band depths. The polynomial fitting was used only to approximate the background smooth component of reflectance, and then subtract it to reveal the absorption features, un-skewed by the background component. Since the background continuum part of reflectance is assumed to be smooth, it can be modeled by a polynomial series. No polynomial fitting was used in the Hapke model. The Hapke algorithm was then used to find the required parameters (n, k, D, ω, *ρ, S*) to simulate the spectra of halite, gypsum and their mixtures. The study also found a favorable comparison between the results of our extracted parameters and those reported in the literature. The input parameters were: incoming angle = 30°, emerging angle = 0°, phase angle g (the angle between the direction of the source to detector) = 30.0°; phase parameters [4] or b = − 0.4, and c = 0.25. We also used B = 0 (g > 15), s = 10^−17^ and S = 1.

**Fig. 5:**
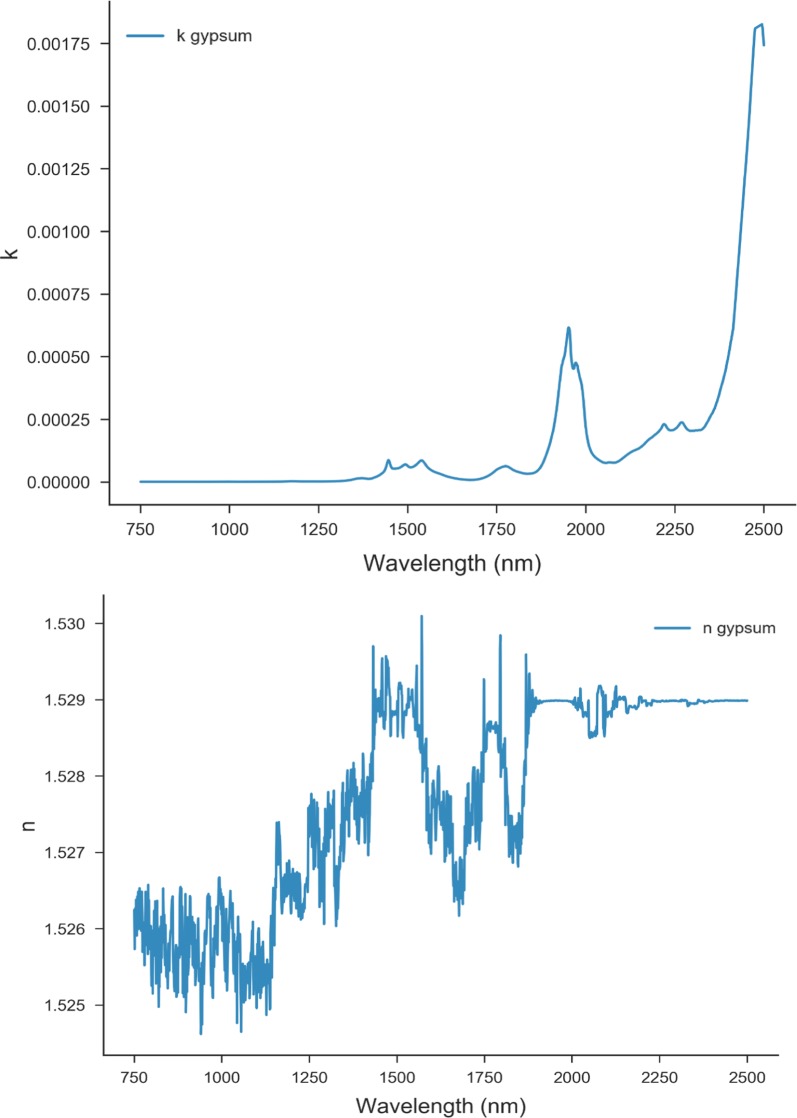
The optimized n and k values for gypsum

**Fig. 6:**
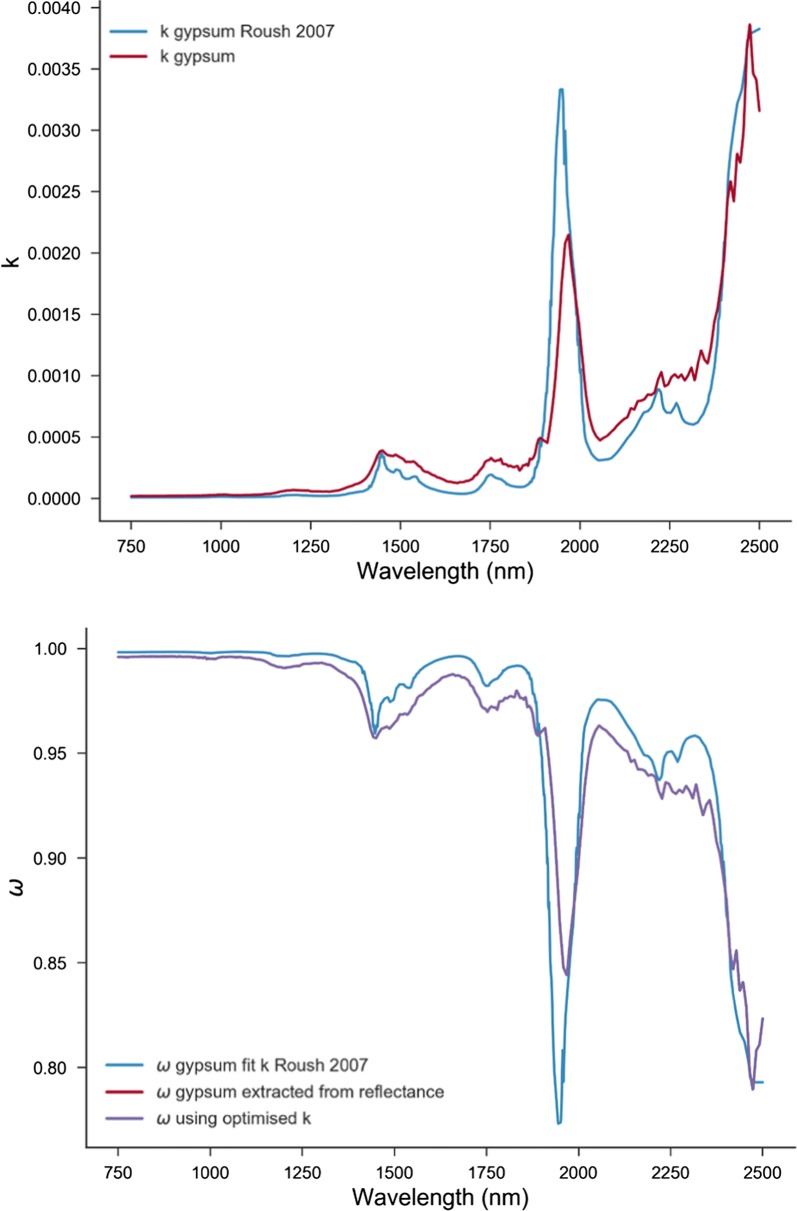
Comparison between the obtained values of k and ω with those reported in the literature

The opposition effect is important only at small phase angles. The macroscopic roughness parameter has the greatest effect at large phase angles. Porosity (filling factor) is unknown. Usually, the determination of the n, k and b and c values would require reflectance spectral measurements of three separate grain sizes at about seven phase angles, g. In addition, if Kramers–Kronig relations are to be used, additional measurements in MIR and UV are necessary [13]. Initially, the n and k values of gypsum available in the literature [12] were used to determine the grain size of the gypsum reflectance spectrum. Then the grain size estimate was used to obtain the optimized spectra of k (Fig. 5). For the halite, suitable k spectra could not be found in the literature, halite having low absorption in NIR. To determine the wavelength dependent imaginary index of refraction a best fit was made simultaneously with D and k. The best fit gives an effective grain size of 26 µm. This value was applied to the reflectance spectra to obtain an improved k spectrum, keeping in mind that the k spectrum could depend on the hydration of the sample [10]. The above procedure significantly improves the fit of the single scattering albedo obtained from reflectance to the one calculated using the optical constants spectra. The comparison between the extracted values of k and ω from fitting and those reported in the literature are demonstrated in Fig. 6, where it appears that the extracted values are comparable at several wavelengths.

**Fig. 7:**
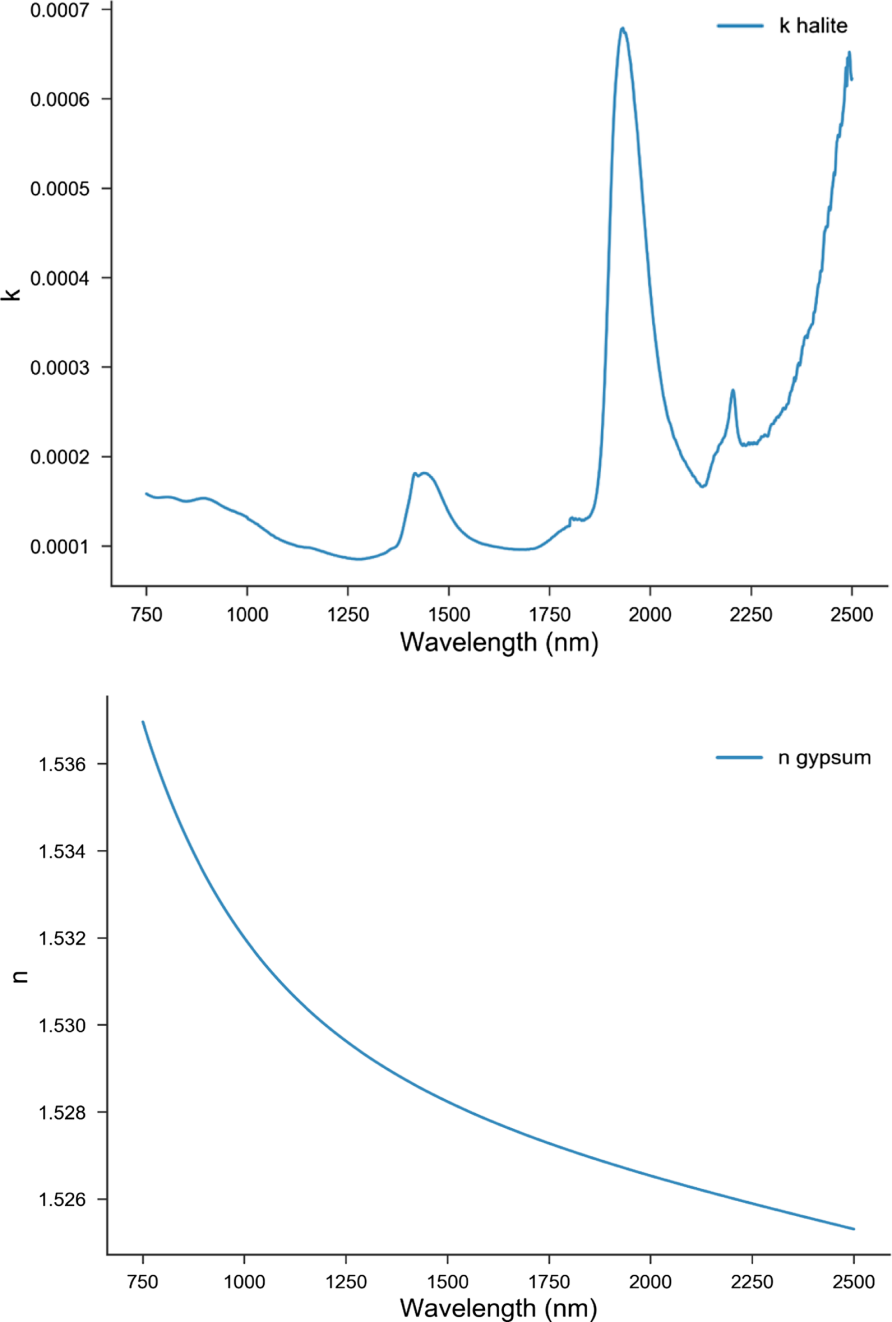
The optimized n and k values for halite

The study followed a similar approach in order to extract n and k for halite (Fig. 7). However, for the real index of refraction (n) of halite the study used the empirical Sellmeier equation. Halite has low absorption in the VIS–NIR and anything else that appears in this region could be related to impurities, therefore, in order to determine the imaginary refractive index of halite a best fit of the bidirectional reflectance was made leaving the grain size parameter free. The best fit results in the halite reference sample having similar grain size to gypsum. For low k reflectance, measurements emphasize the smallest particles [19]. Thus, D values obtained correspond to the smallest particles in the distribution, not the average.

The scattering regime of the two-component system is: (1) for gypsum, single scattering albedo ω is between 0.8 < ω < 0.99, and (2) for halite is close to 1 for the entire region 0.95 < ω < 0.99. For gypsum *α*〈*D*〉 is between 0.01 and 0.11 while gypsum is between 0.1 and 0.5. The region with $\alpha \left\langle D \right\rangle \ll 1$ is the volume scattering region with scattering albedo ω close to 1. The reflectance is dominated by light that has been refracted and transmitted within the volume of the particle. The region of *α*〈*D*〉 < 0.1 is especially susceptible to errors when determining k [19]. This study used the following densities values: *ρ*_*halite*_ = 2.16 g/cm^*3*^, *ρ*_*gypsum*_ = 2.31 g/cm^3^

**Fig. 8:**
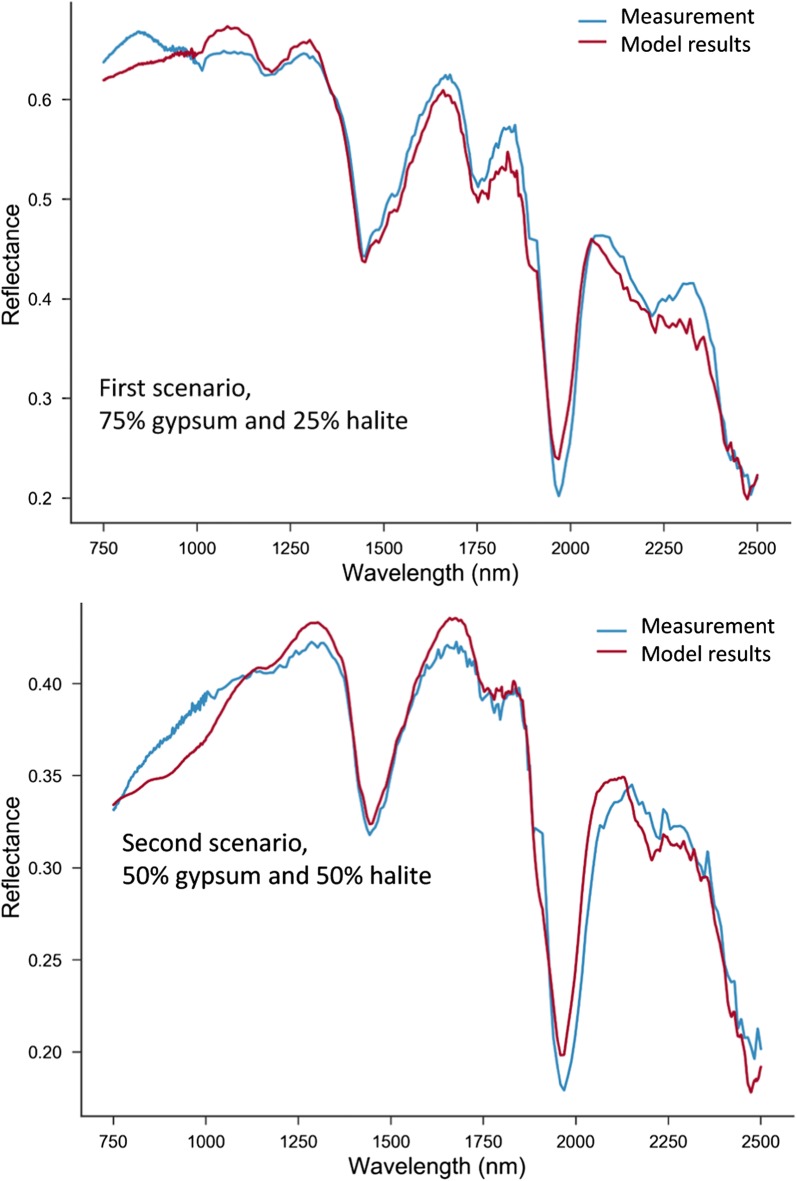
Comparison between the modeled and measured spectra for the first and second mixing scenarios

**Fig. 9:**
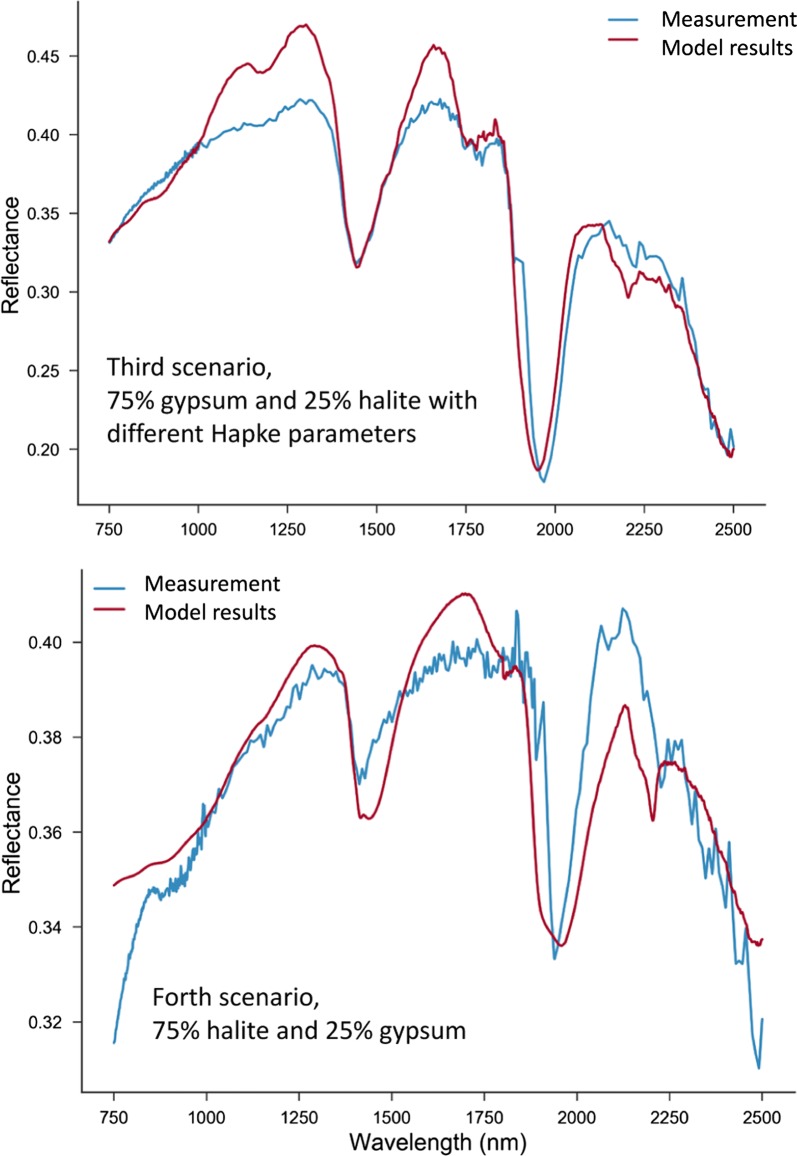
Comparison between the modeled and measured spectra for the third and forth mixing scenarios

To model the spectra, the study used the extracted values from fitting and the common values reported in the literature. For the first scenario, in which the mixture is 75% gypsum and 25% halite, the fitting parameters are: mass fractions or m_gypsum_ = 0.758, m_halite_ = 0.24, grain sizes, D_gypsum_ = 57 µm, D_halite_ = 40.08 µm, and *χ*^2^ = 0.82 (Fig. 8). By comparison, the simulation results for the second mixing scenario, which involves 50% gypsum and 50% halite, we conducted with the following fitting parameters: m_gypsum_ = 0.105, m_halite_ = 0.89, grain sizes D_gypsum_ = 43 µm, D_halite_ = 287 µm, *χ*^2^ = 0.98. The simulation results for these two scenarios are shown in Fig. 8. A third scenario considered the same percentages (i.e. 50% gypsum and 50% halite mixing ratios). The values employed were m_gypsum_ = 0.364, m_halite_ = 0.635, grain sizes, D_gypsum_ = 339 µm, D_halite_ = 46.5 µm, *χ*^2^ = 1.09. For this scenario there was a higher fitting error, as seen in Fig. 9. The fourth and last scenario considered 25% gypsum and 75% halite mixture. The fitting parameters of the mass fractions are m_gypsum_ = 0.0016, m_halite_ = 0.994, grain sizes, D_gypsum_ = 26 µm (fixed), D_halite_ = 265 µm, *χ*^2^ = 0.478. The simulation results for the last two scenarios are shown in Fig. 9. Apart from the last scenario, the results of simulation can be considered satisfactory—the results of the measured and modeled spectra of the first two scenarios almost coincide.

## Conclusions

The approach reported in this contribution was useful for modeling the mixed spectra of gypsum and halite, after obtaining the optical constants n, k for gypsum and halite, and leaving the grain sizes or their ratio as a parameter for fitting. The main challenge facing spectral modeling is that the single scatted albedo depends nontrivially on many variables, including grain sizes, which impact both of the absorption coefficients, and then the fractional cross sections, i.e. there are at least two other reflectance variables which are linked to grain size. The grain size mainly scales the spectra, but there are additional factors as well e.g. porosity factor, and shape of the grains. Although we have measured the spectra from 350 to 2500 nm, we used only the NIR region 750–2500 nm. Impurities make the model unsuitable in the VIS range. The geometry of the measurement is very important for unmixing, since the phase factor cannot be neglected. The study concludes that reflectivity band contrast decreases and becomes overall smaller as particle size increases. High scattering albedo components have larger influence because of the nonlinear dependence of reflectance on it, especially if they are smaller in size. When the absorption is low the sample must be thick (e.g. for halite 100 µm with ω varying only few percent, the sample must be larger than 1 cm).

## Additional files

**Additional file 1.** Paython code and governing equations as well as associated fitting.

**Additional file 2.** Additional details on the proposed fitting method and the used approach to simulate reflectance spectra.

**Additional file 3.** Simulation of reflection spectra for non-intimate linear mixture for comparison.

## Abbreviations

r′: reflectance at wavelength
µ_o_: cosine of the angle of incident light
µ: cosine of the angle of emitted light
g: phase angle
w′: average single scattering albedo
B(g): backscatter function
P(g): average single particle phase function, and
H: Chandrasekhar (1960) H-function for isotropic scatters
