# Supplementary material for: Hapke-based computational method to enable unmixing of hyperspectral data of common salts
Source: Chem Cent J. 2018 Aug 9;12:90. doi: 10.1186/s13065-018-0460-z (PMC6085231; doi:10.1186/s13065-018-0460-z)
Supplement: Supplementary file 1 — Additional file 1. Paython code and governing equations as well as associated fitting. [file 13065_2018_460_MOESM1_ESM.pdf]

Fit_with_nk_02_26_2017

March 12, 2018

```
In [1]: %matplotlib inline
        import pandas as pd
        import numpy as np
        import matplotlib.pyplot as plt
        import pymc as pm
        import scipy as sp
        from scipy.optimize import fsolve, brentq, minimize
        import peakutils
        from scipy.optimize import nnls
        import matplotlib as mpl
        from cycler import cycler
        import lmfit as lm
        mpl.rcParams['axes.prop_cycle'] = cycler(color=['#348ABD', '#A60628', '#7A68A6', '#4678
```

```
In [2]: import seaborn as sns
        #sns.set_style('darkgrid')
        sns.set_style("whitegrid", {"font.family": "serif",   "font.serif": ["Times", "Palatino
        sns.set_context("paper")
        sns.set(style="ticks")
        #flatui = ["#9b59b6", "#3498db", "#95a5a6", "#e74c3c", "#34495e", "#2ecc71"]
        byes = ['#348ABD', '#A60628', '#7A68A6', '#467821','#D55E00',  '#CC79A7', '#56B4E9', '#
        sns.set_palette(byes)
```

0.0.1 Hapke Model

$$r(\mu, \mu_0, g) = \frac{\omega}{4(\mu + \mu_0)} \left\{ (1 + B(g)) P(g) + H(\omega, \mu) H(\omega, \mu_0) - 1 \right\} \frac{S}{\pi}$$

- r reflectance
- ω single-scattering albedo, $(0 \leq \omega(\lambda) \leq 1)$ fraction of the scattered light to incident light by a single particle
- μ_0 cosine of the incident angle
- μ cosine of the emergence angle
- g phase angle
- S macroscopic roughness factor
- B backscattering function
- P particle scattering phase function
- H Chandrasekhar integral function , multiple scattering function

$$P(g) = (1-c)\frac{1-b^2}{(1+2b\cos(g)+b^2)^{3/2}} + c\frac{1-b^2}{(1-2b\cos(g)+b^2)^{3/2}}$$

+ $b(0 \leq \omega(\lambda) \leq 1)$ characterizes the anysotropy of the scattering lobe: $b = 0$ isotropic case, $b = 1$ single direction diffuser + $b(0 \leq \omega(\lambda) \leq 1)$ bacscattering fraction, characterizes the main direction of the diffusion, $c < 0.5$ forward scattering, $c > 0.5$ backward scattering,

$$y = \sqrt{1-\omega}$$

$$H(x) = \left\{1 - [1-y]\,x\left[\left(\frac{1-y}{1+y}\right) + \left(1 - \frac{1}{2}\left(\frac{1-y}{1+y}\right) - x\left(\frac{1-y}{1+y}\right)\right)\ln\left(\frac{1+x}{x}\right)\right]\right\}^{-1}$$

$$B(g) = \frac{B_0}{1 + \frac{1}{h}\tan\left(\frac{g}{2}\right)}$$

- h $(0 \leq h \leq 1)$, angular width, compaction and size distribution
- B_0 $(0 \leq B_0 \leq 1)$, amplitude of the opposition effect, particle transparancy

Isotropic scatterers $P = 1$, no backscattering $B = 0$

$$r(\mu, \mu_0, g) \approx \frac{\omega}{4(\mu+\mu_0)} H(\mu)H(\mu_0)$$

$$H(x) \approx \frac{1}{1 - \omega x\left[r_0 + \frac{1-2r_0x}{2}\ln\left(\frac{1+x}{x}\right)\right]}$$

$r_0 = \frac{1-y}{1+y}$

$$\omega = \frac{4(\mu+\mu_0)r}{H(\mu)H(\mu_0)}$$

Single-scatterig albedo to index of refraction

$$\omega = S_e + (1-S_e)\frac{1-S_i}{1-S_i\Theta}\Theta$$

surface reflection coeficient for externally incident light S_e:

$$S_e = \frac{(n-1)^2+k^2}{(n+1)^2+k^2} + 0.05$$

Reflection coefficient for internally scattered light S_i:

$$S_i = 1.014 - \frac{4}{n(n+1)^2}$$

Particle internal transmission coefficient Θ:

$$\Theta = \frac{r_i + \exp\left(-\sqrt{a(a+s)\langle D\rangle}\right)}{1 + r_i\exp\left(-\sqrt{a(a+s)\langle D\rangle}\right)}$$

Internal bihemispherical reflectance r_i:

$$r_i = \frac{1 - \sqrt{\frac{\alpha}{\alpha+s}}}{1 + \sqrt{\frac{\alpha}{\alpha+s}}}$$

Internal absorption coeficient α:

$$\alpha = \frac{4\pi k}{\lambda}$$

Internal scattering coefficient $S = 0$: Mean free path of photon $\langle D \rangle$:

$$\langle D \rangle = \frac{2}{3}\left[n^2 - \frac{1}{n}(n^2-1)^{\frac{3}{2}}\right] D$$

0.0.2 Mixture Spectra

The single-scattering albedo of a mixture of grains, ω_{mix}, is a linear combination of the single-scattering albedos of its individual endmembers, ω_i:

$$\omega_{mix} = \sum_{i=1}^{N} f_i \omega_i$$

fractional relative cross section of component i:

$$f_i = \frac{\sigma_i}{\sum_{i=1}^{N} \sigma_i}$$

$$\sigma_i = \frac{m_i}{\rho_i D_i}$$

m_i - mass abundance, ρ_i - density, D_i - grain size

```
In [3]: def ch(w,x):
            """
            Calculates Chandrasekhar H function
            w - single scatter albedo
            x = mu or mu_0
            """
            y = np.sqrt(1-w)
            r0 = (1-y) / (1 +y)
            return 1./((1-w*x*(r0 + (1-2*r0*x)/2.*np.log((1+x) / x))))
```

```
In [4]: def reflect(w,mu_0, mu):
            """
            Calculates reflectance of the mixture
            w - single scatter albedo
            mu - cosine of incidence angle
            mu_0 cosine of the emergence angle
            """
            h_mu0 = ch(w, mu_0)
            h_mu = ch(w,mu)
            return w/4./(mu_0 + mu) *h_mu0*h_mu
```

```
In [5]: def wi(n, k, d, lmbda):
            """
            Calculates the single scatter albedo of a component from its optical consants and 
            n - real index of refraction
            k - imaginary index of refraction
            d - grain size
            lmbda - wavelength
            """
            #coeficient of internal attenuation by absorbtion
            alpha = 4*np.pi*k/lmbda
            #coeficient of internal attenuation by scattering
            s = 0.
            # internal difusive reflectance inside a particle
            ri = (1 - np.sqrt(alpha / (alpha + s*np.ones_like(lmbda)))) / (1 + np.sqrt(alpha /
            # average path length of spherical particles of diameter d
            da = 2. / 3. *(n**2 - 1/n *(n**2 - 1)**(3./2.))*d
            # particle internal transmission coefficient
            thta = (ri + np.exp(-np.sqrt(alpha*(alpha + s*np.ones_like(lmbda)))*da)) / (1 + ri
            # Surface reflection coeffecient for externally incident light
            se = ((n-1)**2+k**2) / ((n+1)**2 + k**2) + 0.05*np.ones_like(lmbda)
            #Surface reflection coefficient for internally scattered light
            st = 1.014*np.ones_like(lmbda) - 4./(n*(n+1)**2)
            #albedo component
            return se*np.ones_like(lmbda) + (1-se)*(1-st)/ (np.ones_like(lmbda) - st*thta)*tht

In [6]: def mixspectra(fi,di,n,k,lmbda):
            """
            Calculates the reflectance of a mixture
            fi - vector of relative cross sections
            di - vector of grain sizes
            """
            w = np.zeros_like(lmbda)
            for i in range(len(fi)):
                w = w + fi(i)*wi(n[i],k[i],lmbda)
            return w
```

0.1 Importing data

```
In [7]: # Read gypsum data
        gypsum = pd.read_excel("../Fares/gypsum~1.xls", sheet_name="Sheet1",header
                               = None, names = ['wavelength', 'reflectance'])
        gypsum.set_index("wavelength", inplace=True)

In [8]: gypsum_usga1 = pd.read_excel("../Fares/USGS_Spectral_Library.xlsx", sheet_name="Gypsum
                               = None, names = ['wavelength', 'reflectance'])
        gypsum_usga1.set_index("wavelength", inplace=True)
        gypsum_usga2 = pd.read_excel("../Fares/USGS_Spectral_Library.xlsx", sheet_name="Gypsum
                               = None, names = ['wavelength', 'reflectance'])
```

```
gypsum_usga2.set_index("wavelength", inplace=True)
gypsum_usga3 = pd.read_excel("../Fares/USGS_Spectral_Library.xlsx", sheet_name="Gypsum3
                   = None, names = ['wavelength', 'reflectance'])
gypsum_usga3.set_index("wavelength", inplace=True)
gypsum_usga4 = pd.read_excel("../Fares/USGS_Spectral_Library.xlsx", sheet_name="Gypsum4
                   = None, names = ['wavelength', 'reflectance'])
gypsum_usga4.set_index("wavelength", inplace=True)
gypsum_usga5 = pd.read_excel("../Fares/USGS_Spectral_Library.xlsx", sheet_name="Gypsum5
                   = None, names = ['wavelength', 'reflectance'])
gypsum_usga5.set_index("wavelength", inplace=True)
```

```
In [9]: # Read halite data
        halite = pd.read_excel("../Fares/halite~1.xls", sheet_name="Sheet1",header
                           = None, names = ['wavelength', 'reflectance'])
        halite.set_index("wavelength", inplace=True)
        halite_usga1 = pd.read_excel("../Fares/USGS_Spectral_Library.xlsx", sheet_name="Halite1
                           = None, names = ['wavelength', 'reflectance'])
        halite_usga1.set_index("wavelength", inplace=True)
        halite_usga2 = pd.read_excel("../Fares/USGS_Spectral_Library.xlsx", sheet_name="Halite2
                           = None, names = ['wavelength', 'reflectance'])
        halite_usga2.set_index("wavelength", inplace=True)
        halite_usga3 = pd.read_excel("../Fares/USGS_Spectral_Library.xlsx", sheet_name="Halite3
                           = None, names = ['wavelength', 'reflectance'])
        halite_usga3.set_index("wavelength", inplace=True)
        halite_usga4 = pd.read_excel("../Fares/USGS_Spectral_Library.xlsx", sheet_name="Halite4
                           = None, names = ['wavelength', 'reflectance'])
        halite_usga4.set_index("wavelength", inplace=True)
```

```
In [10]: halite_aster1 = pd.read_excel("../Fares/JPL_Halide.xlsx", sheet_name="0_45um",header
                            = None, names = ['wavelength', 'reflectance'])
         halite_aster1.set_index("wavelength", inplace=True)
         halite_aster2 = pd.read_excel("../Fares/JPL_Halide.xlsx", sheet_name="0_45um_2",heade
                            = None, names = ['wavelength', 'reflectance'])
         halite_aster2.set_index("wavelength", inplace=True)
         halite_aster3 = pd.read_excel("../Fares/JPL_Halide.xlsx", sheet_name="45_125um",heade
                            = None, names = ['wavelength', 'reflectance'])
         halite_aster3.set_index("wavelength", inplace=True)
         halite_aster4 = pd.read_excel("../Fares/JPL_Halide.xlsx", sheet_name="45_125um_2",hea
                            = None, names = ['wavelength', 'reflectance'])
         halite_aster4.set_index("wavelength", inplace=True)
         halite_aster5 = pd.read_excel("../Fares/JPL_Halide.xlsx", sheet_name="125_500um",head
                            = None, names = ['wavelength', 'reflectance'])
         halite_aster5.set_index("wavelength", inplace=True)
         halite_aster6 = pd.read_excel("../Fares/JPL_Halide.xlsx", sheet_name="125_500um_2",he
                            = None, names = ['wavelength', 'reflectance'])
         halite_aster6.set_index("wavelength", inplace=True)
```

```
In [11]: # Read saltsoil data
         saltsoil = pd.read_excel("../Fares/salt-soil-spectra.xls", sheet_name="Sheet1",header
```

```
                        = 0)
        saltsoil.set_index("Wavelength", inplace=True)
```

```
In [12]: # normalize refrence the values
         gypsum_ref = gypsum.reflectance.values / 100.
         halite_ref = halite.reflectance.values
```

```
In [13]: def plotSpectrum(x = [], y = [], label =[], xlabel = "", ylabel = "",  save =  False,
             fig, ax = plt.subplots(figsize=(8,6))
             ax.spines['top'].set_visible(False)
             ax.spines['right'].set_visible(False)
             ax.xaxis.set_tick_params(top='off', direction='out', width=1)
             ax.yaxis.set_tick_params(right='off', direction='out', width=1)
             for i in range(len(y)):
                 plt.plot(x[i], y[i], label=label[i])
             plt.legend()
             plt.xlabel(xlabel, fontsize = 16)
             plt.ylabel(ylabel, fontsize = 16)
             plt.tick_params(labelsize = 12)
             plt.tight_layout()
             #plt.title("gypsum reference")
             if save:
                 plt.savefig(savefile, dpi=300)
             return fig
```

```
In [14]: gypsum_ref_fig = plotSpectrum([gypsum.index.values,
                                        gypsum_usga1.index.values*1000,
                                        gypsum_usga2.index.values*1000,
                                        gypsum_usga3.index.values*1000,
                                        gypsum_usga4.index.values*1000],
                                       [gypsum_ref,
                                       gypsum_usga1.reflectance.values,
                                       gypsum_usga2.reflectance.values,
                                       gypsum_usga3.reflectance.values,
                                       gypsum_usga4.reflectance.values],
                                       ["gypsum reference",
                                       "gypsum usga1," + " $D < $" + " 5 $\mu$m",
                                       "gypsum usga2," + " $D < $" + " 74 $\mu$m",
                                       "gypsum usga3," + " 74 $\mu$m <" + "$ D < $" + " 250 $\
                                       "gypsum usga4," + " $ D > $" + " 250 $\mu$m"],
                                       "Wavelength (nm)", "Reflectance",False,"./figs/gypsum_r
```

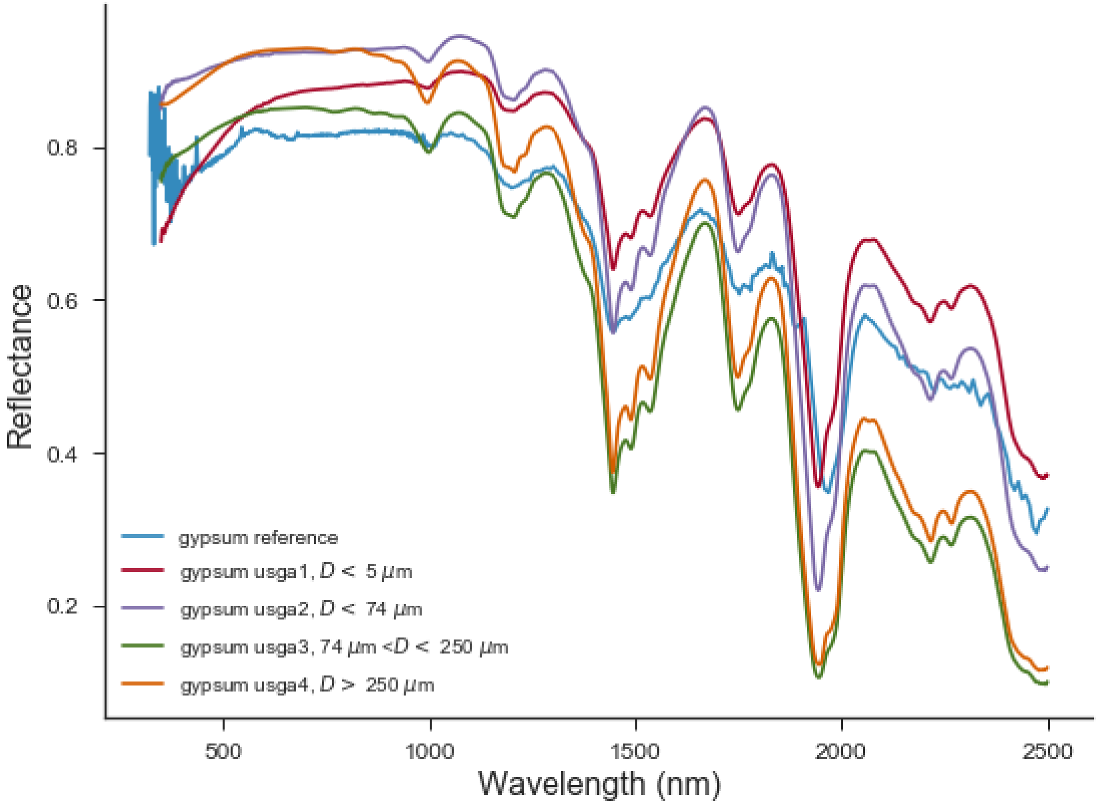

In [15]:
```
# halite reference data
fig, ax = plt.subplots(figsize=(8,6))
ax.spines['top'].set_visible(False)
ax.spines['right'].set_visible(False)
ax.xaxis.set_tick_params(top='off', direction='out', width=1)
ax.yaxis.set_tick_params(right='off', direction='out', width=1)
plt.plot(halite_aster1.index.values*1000, halite_aster1.reflectance.values/100., label
plt.plot(halite_aster2.index.values*1000, halite_aster2.reflectance.values/100., label
plt.plot(halite_aster4.index.values*1000, halite_aster4.reflectance.values/100., label
plt.plot(halite_aster6.index.values*1000, halite_aster6.reflectance.values/100., label
#plt.grid()
plt.legend()
plt.xlabel("Wavelength (nm)", fontsize = 16)
plt.ylabel("Reflectance", fontsize = 16)
plt.tick_params(labelsize = 12)
plt.tight_layout()
#plt.title("gypsum reference")
#plt.savefig("./figs/halite_reference_spectra.png", dpi=300)
```

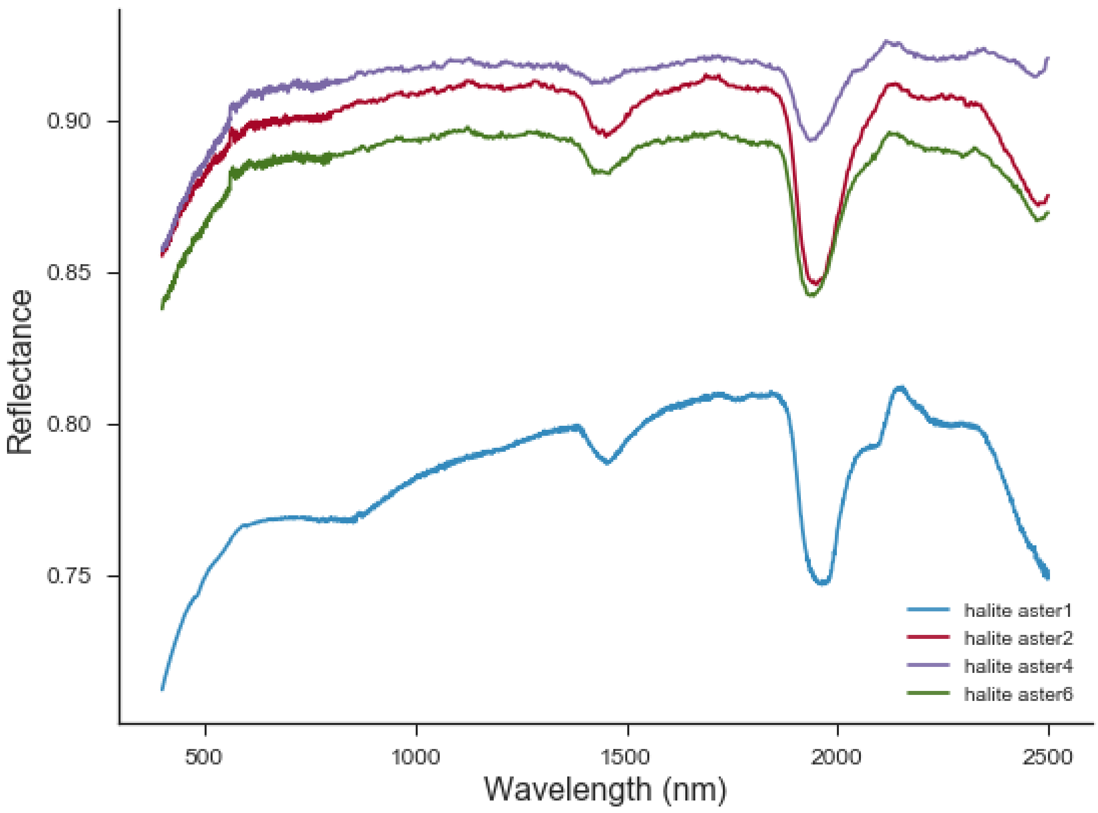


```
In [16]: # scaled gypsum reference data
         fig, ax = plt.subplots(figsize=(8,6))
         ax.spines['top'].set_visible(False)
         ax.spines['right'].set_visible(False)
         ax.xaxis.set_tick_params(top='off', direction='out', width=1)
         ax.yaxis.set_tick_params(right='off', direction='out', width=1)
         plt.plot(gypsum.index.values, gypsum_ref/ gypsum_ref.max(), label="gypsum reference")
         plt.plot(gypsum_usga1.index.values*1000, gypsum_usga1.reflectance.values/ gypsum_usga
         plt.plot(gypsum_usga2.index.values*1000, gypsum_usga2.reflectance.values/ gypsum_usga
         plt.plot(gypsum_usga3.index.values*1000, gypsum_usga3.reflectance.values/ gypsum_usga
         plt.plot(gypsum_usga4.index.values*1000, gypsum_usga4.reflectance.values/ gypsum_usga
         #plt.grid()
         plt.legend()
         plt.xlabel("Wavelength (nm)", fontsize = 16)
         plt.ylabel("Scaled Reflectance", fontsize = 16)
         plt.tick_params(labelsize = 12)
         plt.tight_layout()
         #plt.title("gypsum reference")
         #plt.savefig("./figs/scaled_gypsum_reference_spectra.png", dpi=300)
```

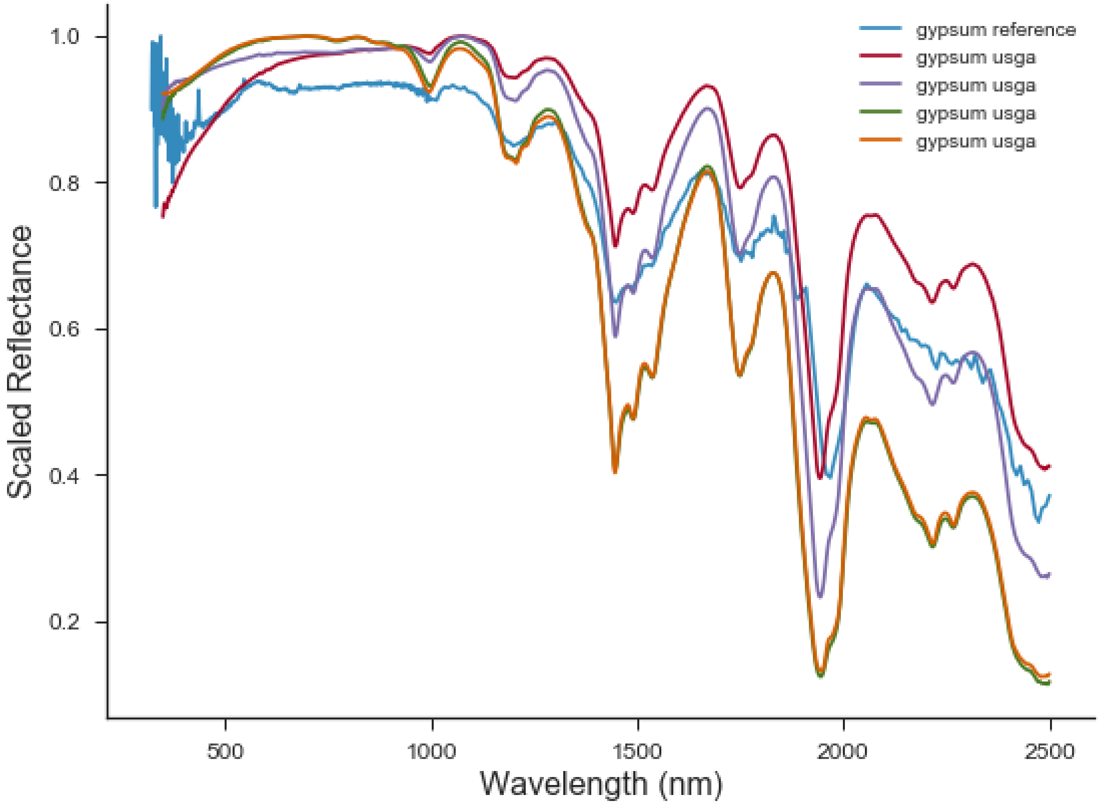


```
In [58]: # scaled halite reference data
         fig, ax = plt.subplots(figsize=(8,6))
         ax.spines['top'].set_visible(False)
         ax.spines['right'].set_visible(False)
         ax.xaxis.set_tick_params(top='off', direction='out', width=1)
         ax.yaxis.set_tick_params(right='off', direction='out', width=1)
         plt.plot(halite.index.values, halite_ref/ halite_ref.max(), label="halite reference")
         plt.plot(halite_usga1.index.values*1000, halite_usga1.reflectance.values/ halite_usga
         plt.plot(halite_usga2.index.values*1000, halite_usga2.reflectance.values/ halite_usga
         plt.plot(halite_usga3.index.values*1000, halite_usga3.reflectance.values/ halite_usga
         plt.plot(halite_usga4.index.values*1000, halite_usga4.reflectance.values/ halite_usga
         #plt.grid()
         plt.legend()
         plt.xlabel("Wavelength (nm)", fontsize = 16)
         plt.ylabel("Scaled Reflectance", fontsize = 16)
         plt.tick_params(labelsize = 12)
         plt.tight_layout()
         #plt.title("gypsum reference")
         #plt.savefig("./figs/scaled_halite_reference_spectra.png", dpi=300)
```

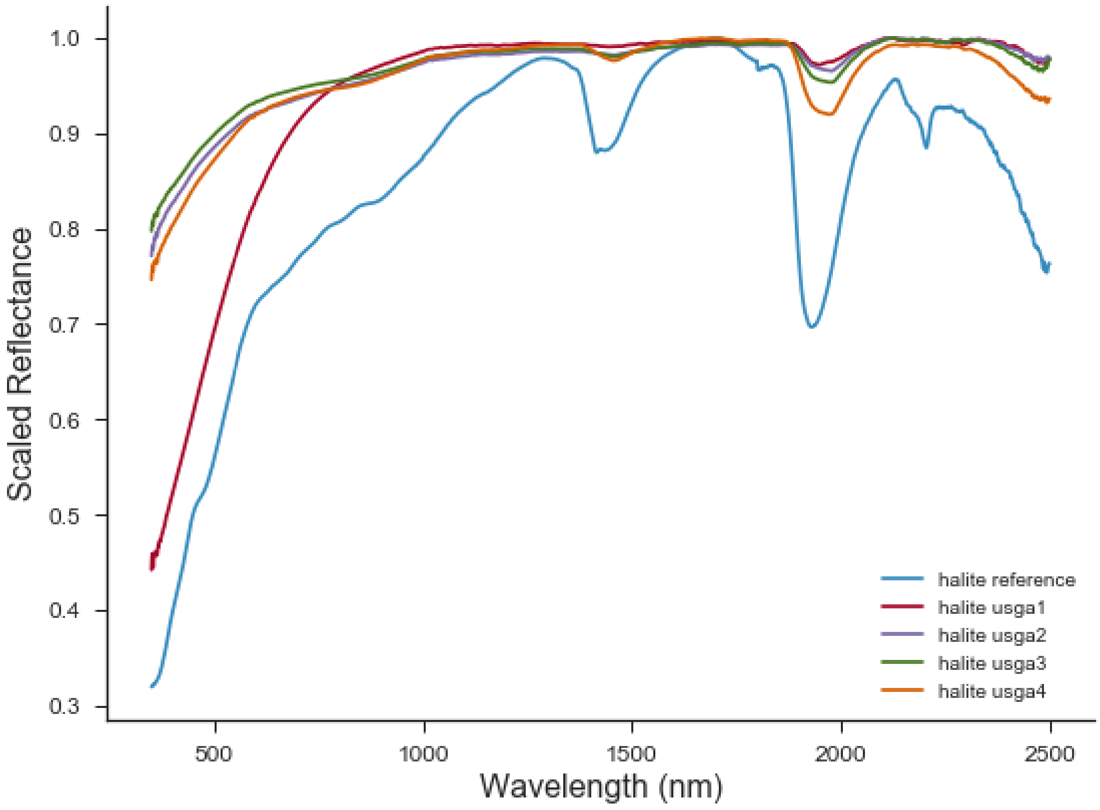


```
In [18]: # mixed soild data
         salfig = saltsoil.loc[:,['HR 0.75G: 0.25H', 'HR 0.5 G:0.5H',
                                  'HR0.25G:0.75H']].plot(title = "data", fontsize = 14)
         ax = salfig.axes
         fig = salfig.get_figure()
         ax.spines['top'].set_visible(False)
         ax.spines['right'].set_visible(False)
         ax.xaxis.set_tick_params(top='off', direction='out', width=1)
         ax.yaxis.set_tick_params(right='off', direction='out', width=1)
         fig.set_size_inches((8,6))
         salfig.set_xlabel("Wavelength (nm)", fontsize = 16)
         salfig.set_ylabel("Reflectance", fontsize = 16)
         ax.tick_params(labelsize = 12)
         fig.tight_layout()
         #fig.savefig("./figs/data.png", dpi =300)
```

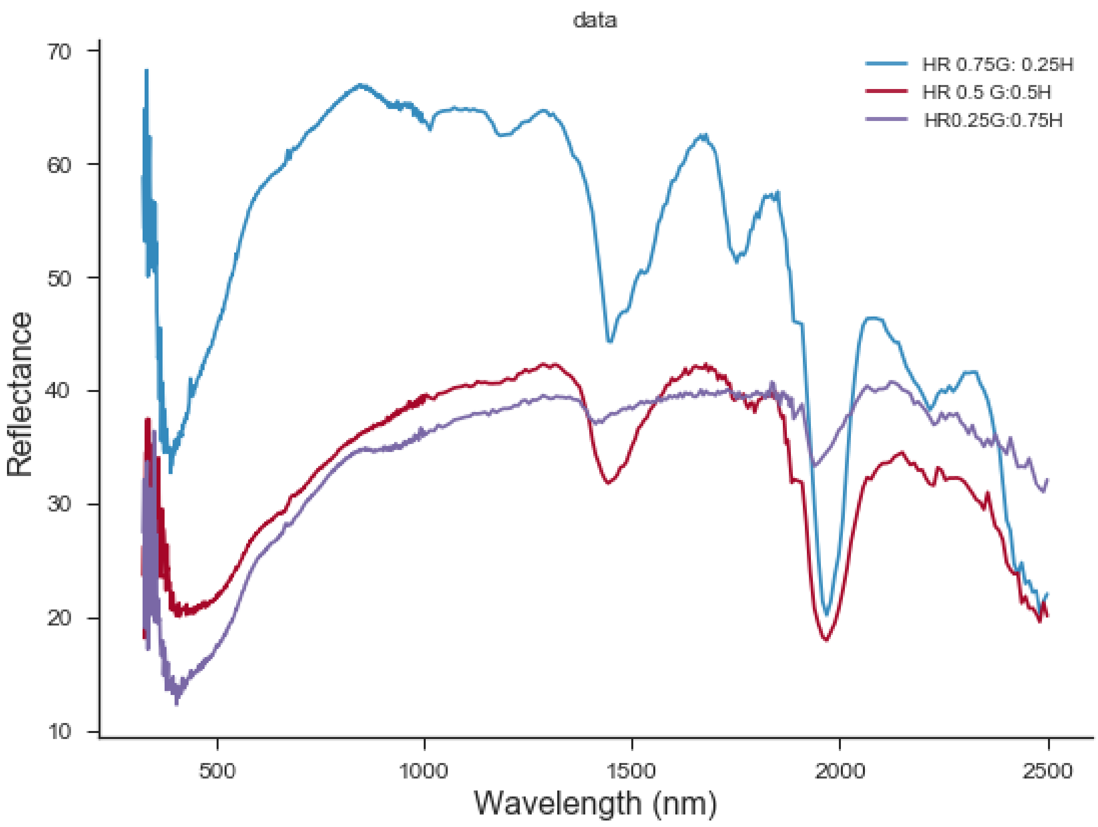

0.2 Interpolate data to have same dimensions

```
In [19]: # interpolate the two referece to have the same dimensions
         wavelengths = np.linspace(322,2500,1000)
         rh = np.interp(wavelengths,halite.index.values, halite_ref)
         rg = np.interp(wavelengths,gypsum.index.values, gypsum_ref)

In [20]: # interpolate the data to have the same dimensions
         g075h025 = saltsoil["HR 0.75G: 0.25H"]
         g05h05 = saltsoil["HR 0.5 G:0.5H"]
         g025h075 = saltsoil["HR0.25G:0.75H"]
         saltsoil_wavelengths = saltsoil.index.values
         r_g075h025 = np.interp(wavelengths,saltsoil_wavelengths, g075h025.values) /100.
         r_g05h05 = np.interp(wavelengths,saltsoil_wavelengths, g05h05.values) /100.
         r_g025h075 = np.interp(wavelengths,saltsoil_wavelengths, g025h075.values) /100.

In [21]: a = np.interp(wavelengths,halite_aster2.index.values*1000,halite_aster2.reflectance.va

In [22]: plt.plot(wavelengths,a)

Out[22]: [<matplotlib.lines.Line2D at 0x2b0d65d39b0>]
```

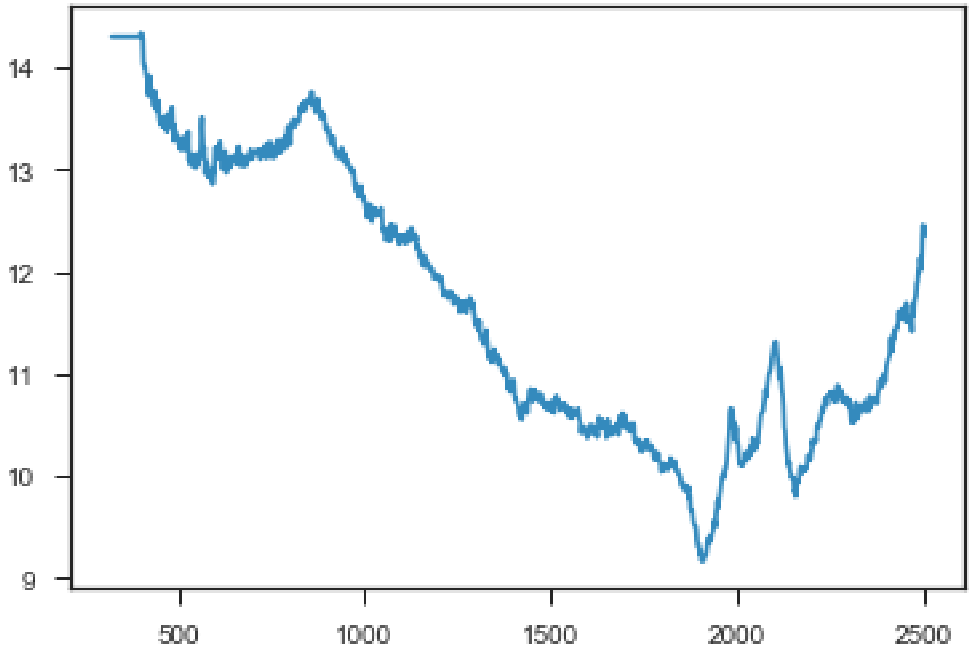


```
In [23]: #exclude the UV noisy part
         wavelengths = wavelengths[35:]
         rh = rh[35:]
         rg = rg[35:]
         r_g075h025 = r_g075h025[35:]
         r_g05h05 = r_g05h05[35:]
         r_g025h075 = r_g025h075[35:]
```

```
In [24]: # plot data and reference
         fig, ax = plt.subplots(figsize=(8,6))
         ax.spines['top'].set_visible(False)
         ax.spines['right'].set_visible(False)
         ax.xaxis.set_tick_params(top='off', direction='out', width=1)
         ax.yaxis.set_tick_params(right='off', direction='out', width=1)
         plt.plot(wavelengths,rh, label="halite reference")
         plt.plot(wavelengths,rg, label="gypsum reference")
         plt.plot(wavelengths,r_g075h025, label="H0.25 G0.75")
         plt.plot(wavelengths,r_g05h05, label="H0.5 G0.5")
         plt.plot(wavelengths,r_g025h075, label="H0.75 G0.25")
         #plt.grid()
         plt.legend()
         plt.xlabel("Wavelength (nm)", fontsize = 16)
         plt.ylabel("Reflectance", fontsize = 16)
         plt.tick_params(labelsize = 12)
         plt.tight_layout()
```

```
#plt.title("gypsum reference")
#plt.savefig("./figs/data_and_reference_spectra.png", dpi=300)
```

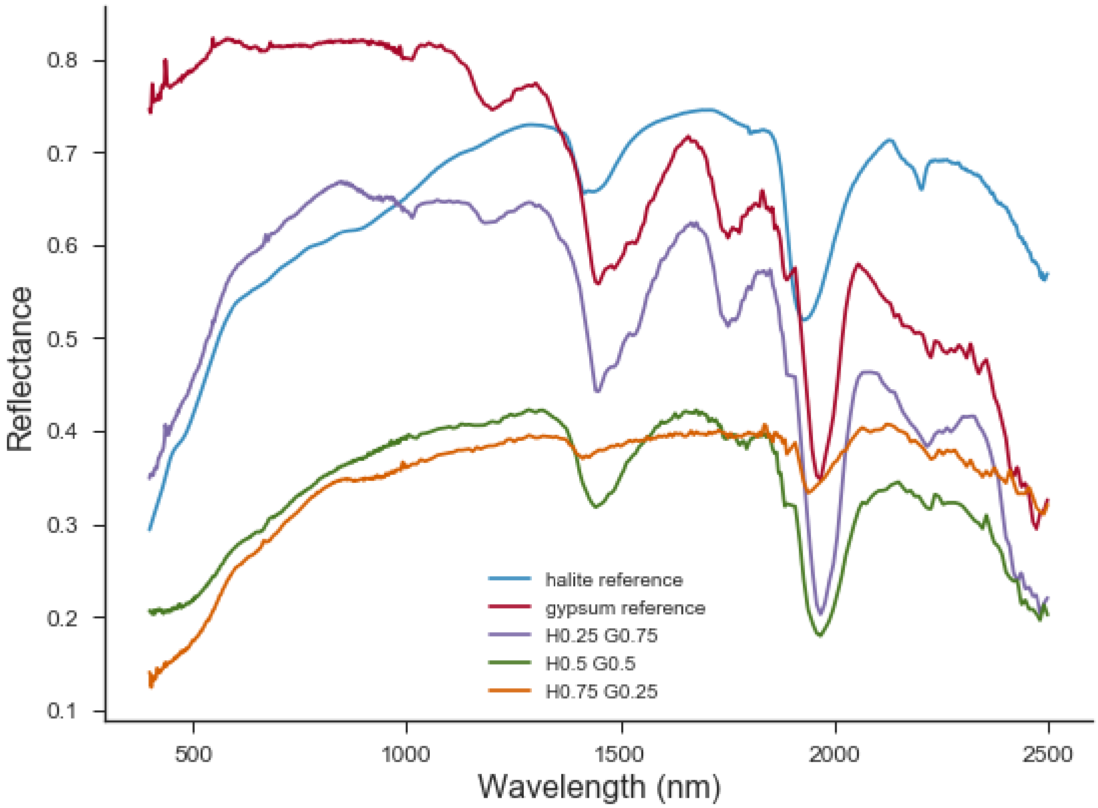

In [25]:
```
# plot data and reference
fig, ax = plt.subplots(figsize=(8,6))
ax.spines['top'].set_visible(False)
ax.spines['right'].set_visible(False)
ax.xaxis.set_tick_params(top='off', direction='out', width=1)
ax.yaxis.set_tick_params(right='off', direction='out', width=1)
plt.plot(wavelengths,rh / rh.max(), label="halite reference")
plt.plot(wavelengths,rg / rg.max(), label="gypsum reference")
plt.plot(wavelengths,r_g075h025 / r_g075h025.max(), label="H0.25 G0.75")
plt.plot(wavelengths,r_g05h05 / r_g05h05.max(), label="H0.5 G0.5")
plt.plot(wavelengths,r_g025h075 / r_g025h075.max(), label="H0.75 G0.25")
#plt.grid()
plt.legend()
plt.xlabel("Wavelength (nm)", fontsize = 16)
plt.ylabel("Scaled Reflectance", fontsize = 16)
plt.tick_params(labelsize = 12)
plt.tight_layout()
#plt.title("gypsum reference")
#plt.savefig("./figs/scaled_data_and_reference_spectra.png", dpi=300)
```

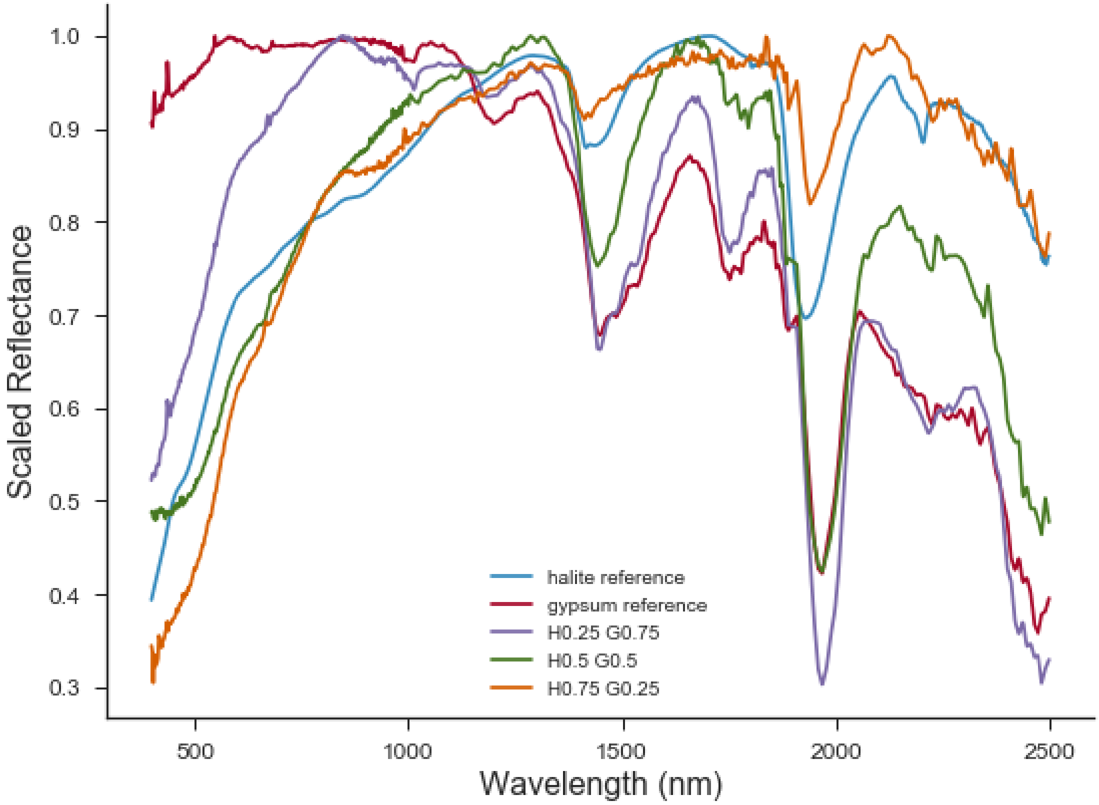

Extracting ssa ω

```
In [26]: print(plt.style.available)

['bmh', 'classic', 'dark_background', 'fast', 'fivethirtyeight', 'ggplot', 'grayscale', 'seabo
```

```
In [27]: # solve for w in reflection function
         # function to minimize
         incoming_angle = 0.0
         emmerging_angle = 30.0
         def residual_reflect(w,r):
             """
             Minimization fitting function for extracting w values
             params : w, inc_angle, emr_angle
             r : reflectance data to subtract
             """
             mu0 = np.cos(incoming_angle*np.pi/180);
             mu = np.cos(emmerging_angle*np.pi/180);
             return reflect(w,mu0,mu) - r
         def extract_ssa(r_data):
```

```
    w = np.zeros_like(r_data)
    for i in range(len(r_data)):
        w[i] = brentq(residual_reflect, 0., 1, args=(r_data[i]))
    return w
```

In [28]:
```
# extract ssa for gipsum by nonlin fitting the reflectance sample
wg = extract_ssa(rg)
wh = extract_ssa(rh)
```

In [29]:
```
 ssa_reference_fig = plotSpectrum([wavelengths,
                                   wavelengths],
                                  [wg,
                                  wh],
                                  ["ssa gypsum",
                                  "ssa halite"],
                                  "Wavelength (nm)", "SSA",False,"./figs/sa_references.png
```

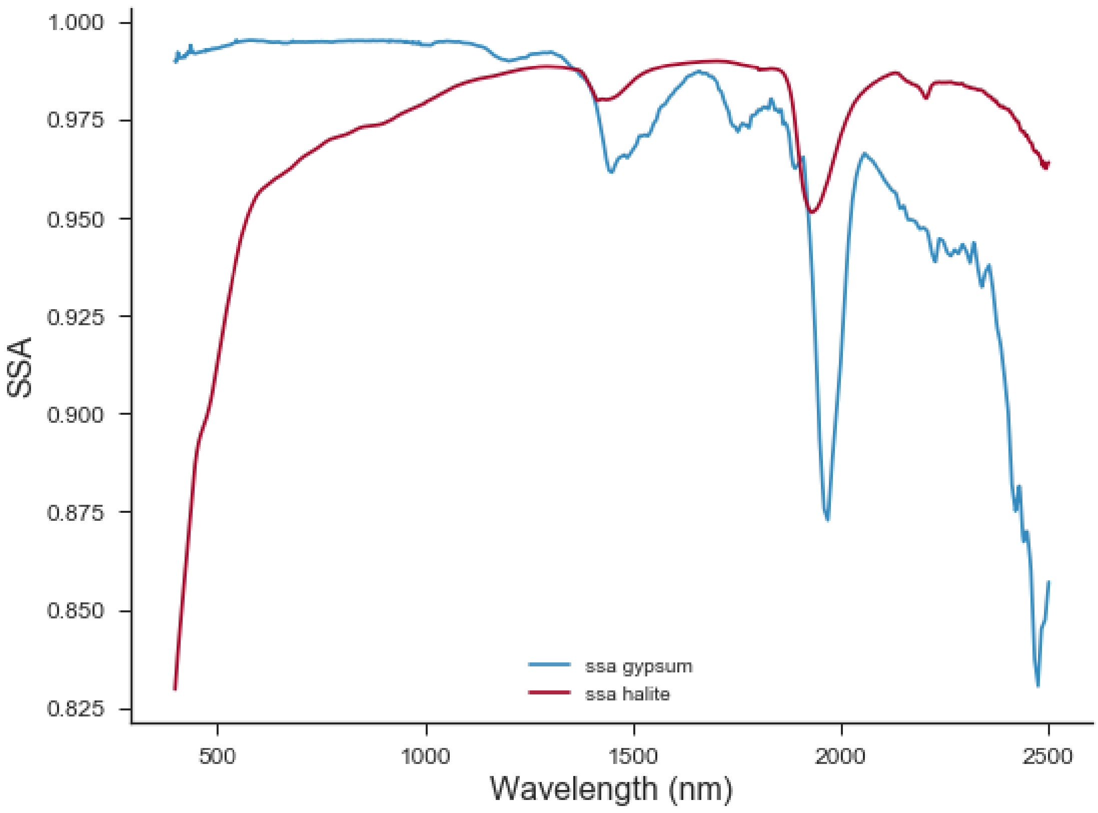

In [30]:
```
# Get ssa for slatsoil
wg075h025 = extract_ssa(r_g075h025)
wg05h05 = extract_ssa(r_g05h05)
wg025h075 = extract_ssa(r_g025h075)
```

```
In [31]: ssa_soil_fig = plotSpectrum([wavelengths,
                                      wavelengths,
                                      wavelengths],
                                     [wg075h025,
                                      wg05h05,
                                      wg025h075],
                                     ["ssa H0.25 G0.75",
                                     "ssa H0.5 G0.5",
                                     "ssa H0.75 G0.25"],
                                     "Wavelength (nm)", "SSA",False,"./figs/ssa_data.png")
```

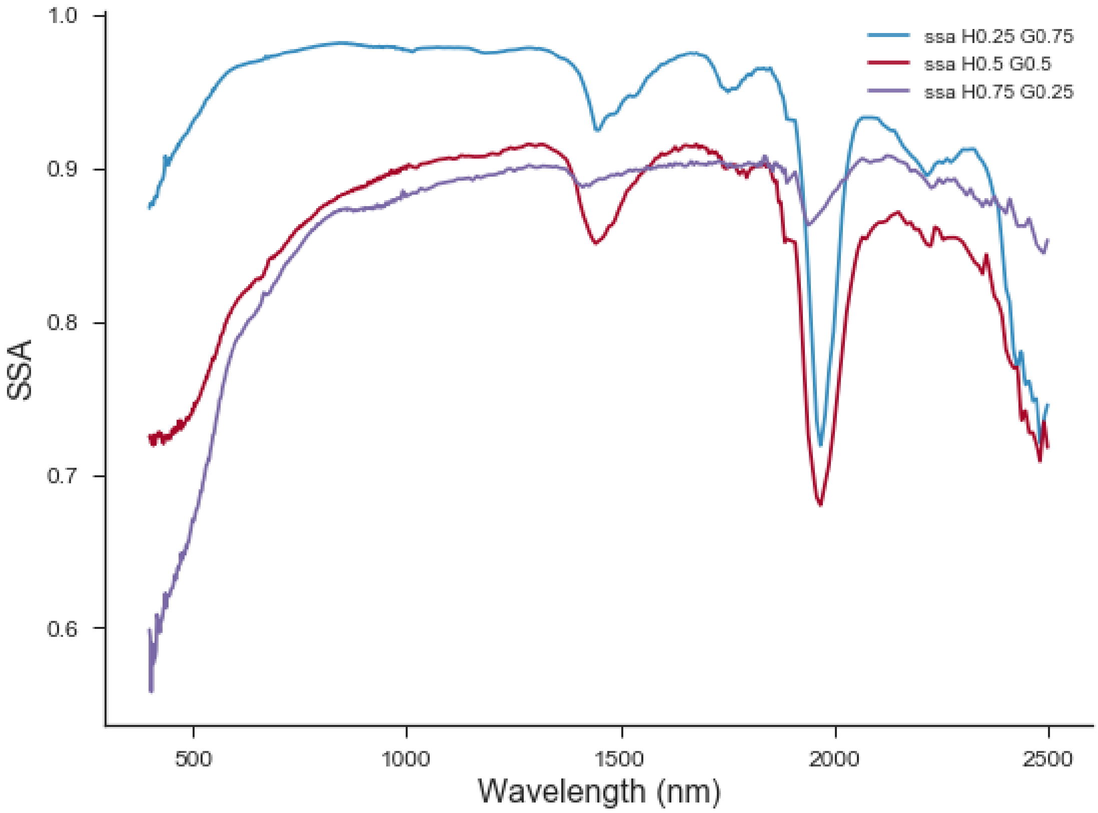

0.3 Determining SSA from n and k

```
In [32]: gypsum_n = pd.read_excel("../Fares/gypsum_nk.xlsx", sheet_name="n",header
                        = None, names = ['wavelength', 'n'])
         gypsum_n.set_index("wavelength", inplace=True)
         gypsum_k = pd.read_excel("../Fares/gypsum_nk.xlsx", sheet_name="k",header
                        = None, names = ['wavelength', 'k'])
         gypsum_k.set_index("wavelength", inplace=True)
```

```
In [33]: g_n = np.interp(wavelengths, gypsum_n.index.values*1000, gypsum_n.n.values)
         g_k = np.interp(wavelengths, gypsum_k.index.values*1000, gypsum_k.k.values)
```

```
In [34]: gypsum_k_fig= plotSpectrum([wavelengths], [g_k], ['k gypsum'], "Wavelength (nm)", "k"
```

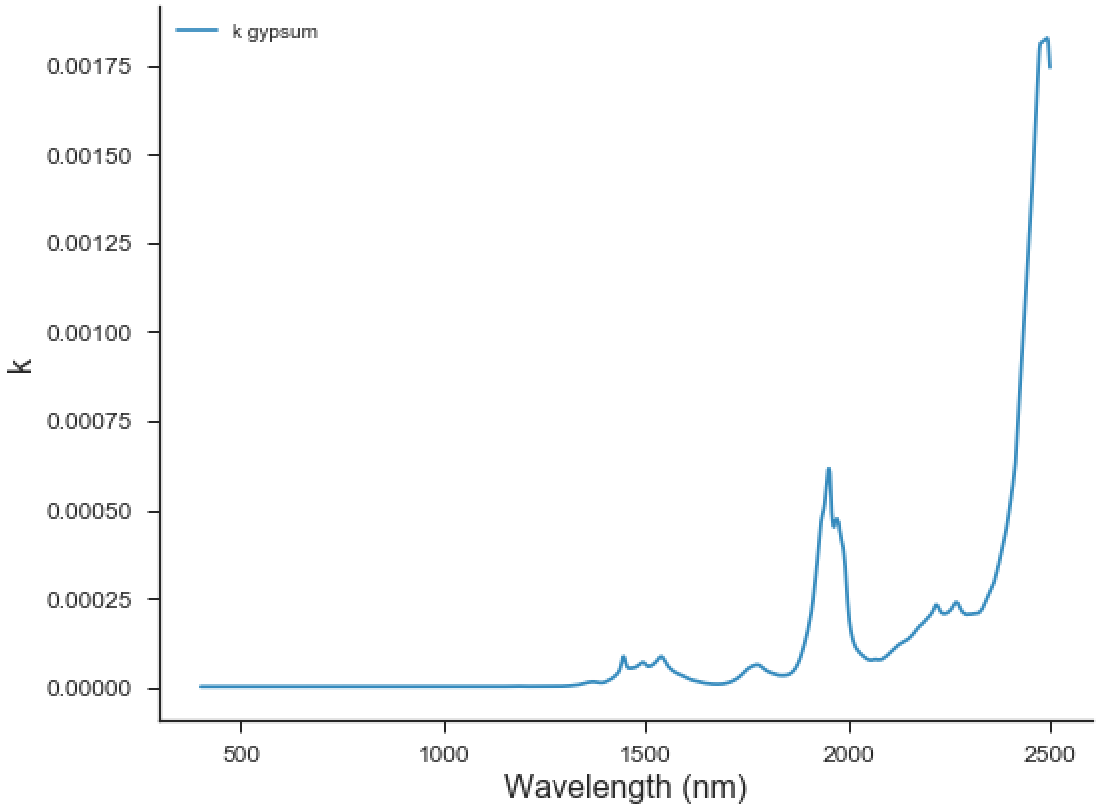


```
In [35]: gypsum_n_fig= plotSpectrum([wavelengths], [g_n], ['n gypsum'], "Wavelength (nm)", "n"
```

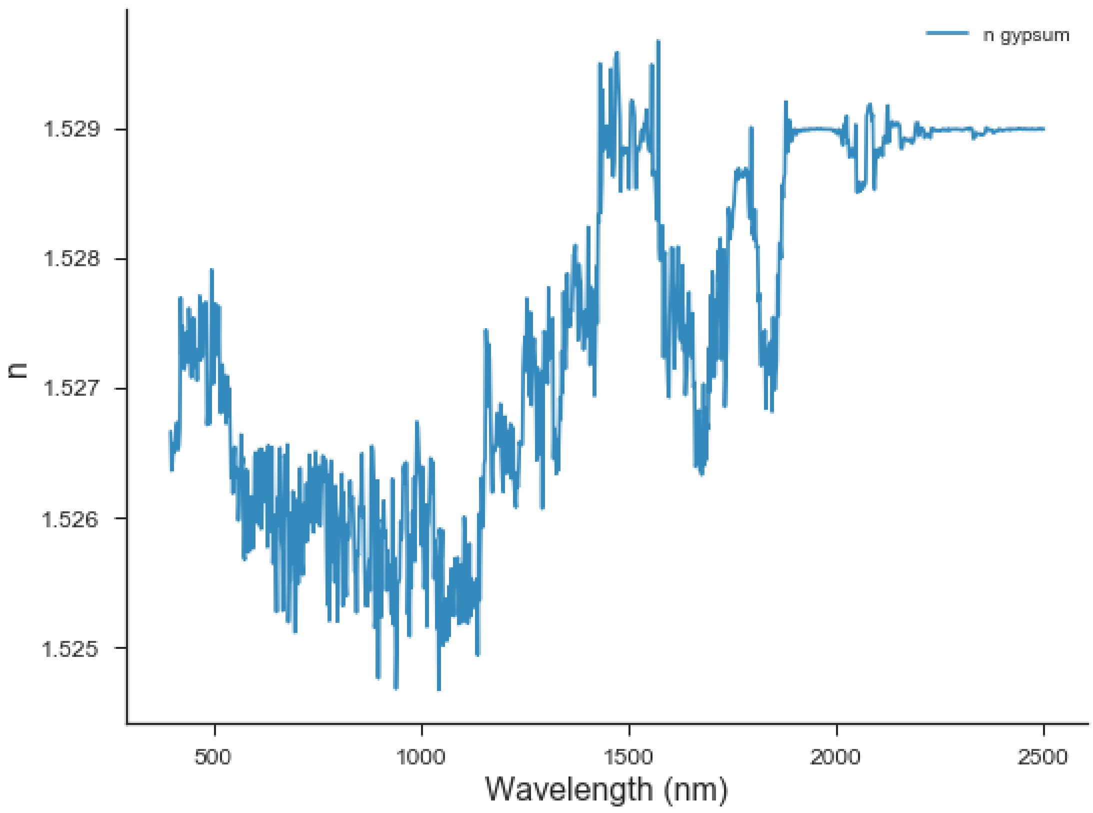


```
In [38]: def residual_grainSize(d):
             ss = np.sqrt((wi(g_n, g_k, d, wavelengths) - wg)**2)
             return ss.sum()
         ans = minimize(residual_grainSize, 0, method='nelder-mead', options={'xtol': 1e-8, 'di
         print(ans.x)
```

```
Optimization terminated successfully.
         Current function value: 11.606832
         Iterations: 74
         Function evaluations: 148
[ 21754.60825139]
```

```
In [40]: d_grain = ans.x[0]
         print(d_grain)
```

```
21754.6082514
```

```
In [44]: def residual_k(k):
             ss = np.sqrt((wi(g_n[10], k, d_grain, wavelengths[10]) - wg[10])**2)
             return ss.sum()
```

```
ansk = minimize(residual_k, 0, method='nelder-mead', options={'xtol': 1e-8, 'disp': T
print(ansk.x)
```

```
Optimization terminated successfully.
         Current function value: 0.000005
         Iterations: 20
         Function evaluations: 41
[  6.59179688e-06]
```

```
C:\ProgramData\Anaconda3\lib\site-packages\ipykernel_launcher.py:14: RuntimeWarning: invalid va
```

```
In [47]: def residual_kk(k,n,d,lbda,w):
             ss = np.sqrt((wi(n, k, d, lbda) - w)**2)
             return ss.sum()
         anskk = minimize(residual_kk, 0, args = (g_n[10],d_grain,wavelengths[10],wg[10]), meth
         print(anskk.x)
```

```
Optimization terminated successfully.
         Current function value: 0.000005
         Iterations: 20
         Function evaluations: 41
[  6.59179688e-06]
```

```
C:\ProgramData\Anaconda3\lib\site-packages\ipykernel_launcher.py:14: RuntimeWarning: invalid va
```

```
In [49]: k_m = []
         for i in range(len(wavelengths)):
             anskk = minimize(residual_kk, 0, args = (g_n[i],d_grain,wavelengths[i],wg[i]), me
             k_m.append(anskk.x[0])
```

```
C:\ProgramData\Anaconda3\lib\site-packages\ipykernel_launcher.py:14: RuntimeWarning: invalid va
```

```
In [79]: g_k_min = np.array(k_m)
```

```
In [55]: k_min_fig = plotSpectrum([wavelengths, wavelengths], [g_k_min, g_k], ['k from r', "k"]
```

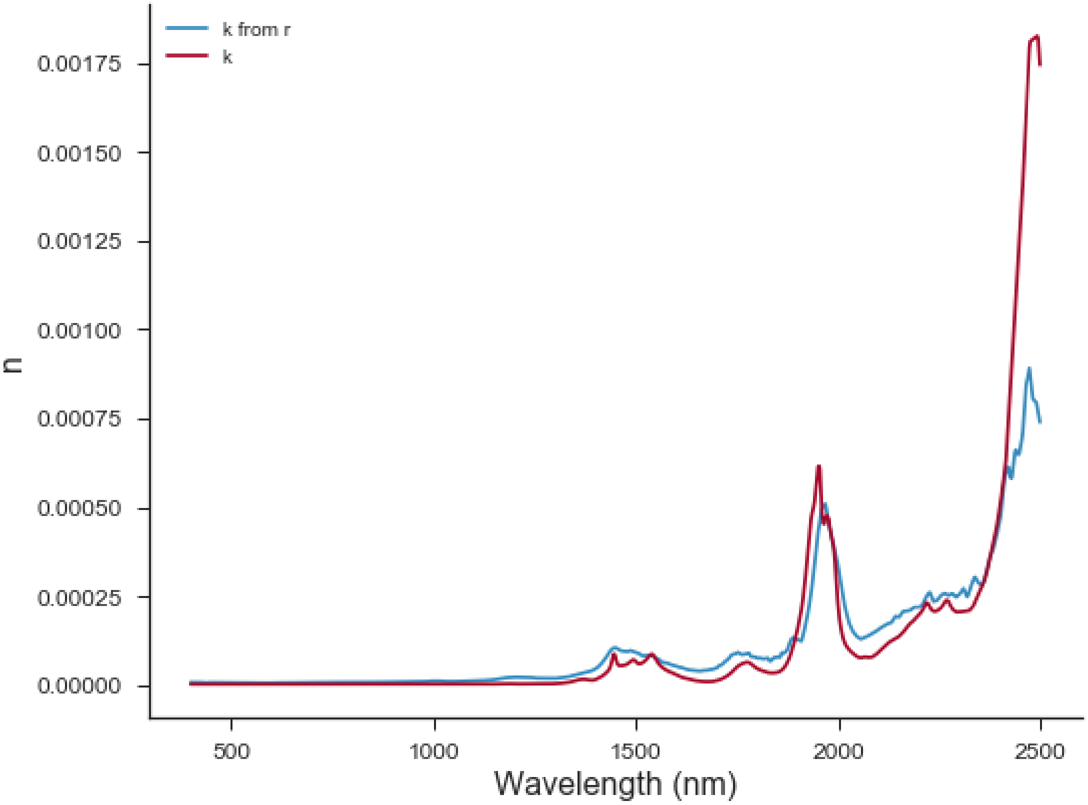


```
In [56]: ssa_gypsum_nk= wi(g_n, g_k, d_grain, wavelengths)
         ssa_gypsum_nk2= wi(g_n, k_min, d_grain, wavelengths)

In [57]: # plot data and reference
         fig, ax = plt.subplots(figsize=(8,6))
         ax.spines['top'].set_visible(False)
         ax.spines['right'].set_visible(False)
         ax.xaxis.set_tick_params(top='off', direction='out', width=1)
         plt.plot(wavelengths, ssa_gypsum_nk, label='ssa from nk and <D>' + ", <D> = " + str(14
         plt.plot(wavelengths, ssa_gypsum_nk2, label='ssa from nk and <D>' + ", <D> = " + str(a
         plt.plot(wavelengths, wg, label='ssa gypsum')
         #plt.grid()
         plt.legend()
         plt.xlabel("Wavelength (nm)", fontsize = 16)
         plt.ylabel("SSA", fontsize = 16)
         plt.tick_params(labelsize = 12)
         plt.tight_layout()
         #plt.title("gypsum reference")
         #plt.savefig("./figs/fitt_ssa_gypsum.png", dpi=300)
```

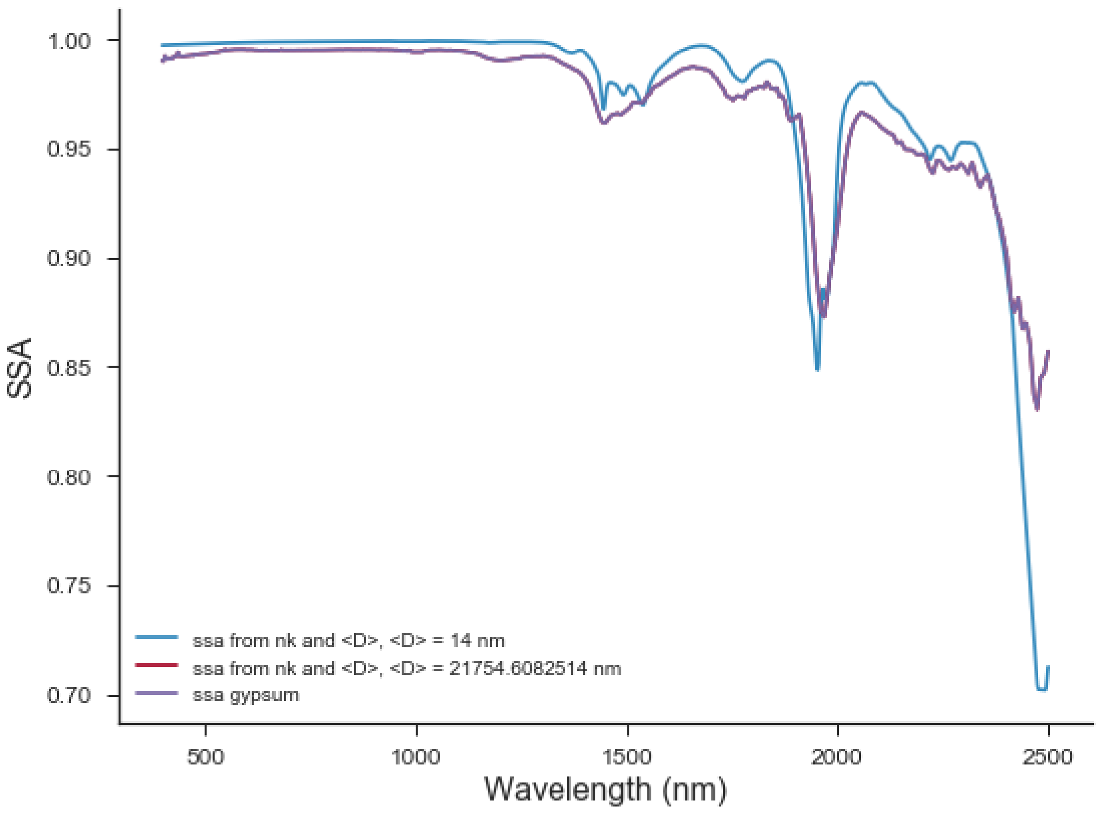

0.4 Halite n and k

```
In [191]: h_n = 1.5443*np.ones_like(wavelengths)

In [252]: k_h_m = []
          for i in range(len(wavelengths)):
              anskk = minimize(residual_kk, 0, args = (h_n[i],24000,wavelengths[i],wh[i]), met
              k_h_m.append(anskk.x[0])

C:\ProgramData\Anaconda3\lib\site-packages\ipykernel_launcher.py:14: RuntimeWarning: invalid va



In [253]: h_k_min = np.array(k_h_m)

In [347]: k_h_min_fig = plotSpectrum([wavelengths], [h_k_min], ['k from r halite '], "Waveleng
```

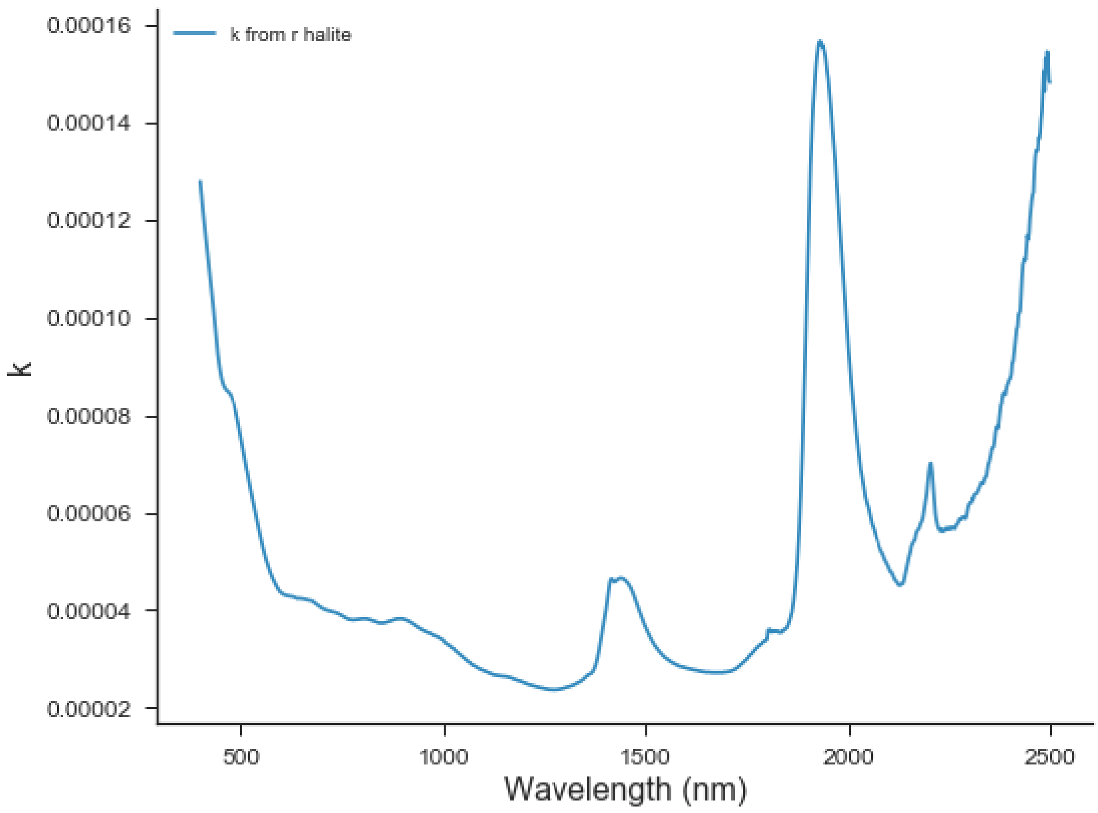


```
In [255]: ssa_halite_nk= wi(h_n, h_k_min, d_grain, wavelengths)

In [256]: # plot data and reference
          fig, ax = plt.subplots(figsize=(8,6))
          ax.spines['top'].set_visible(False)
          ax.spines['right'].set_visible(False)
          ax.xaxis.set_tick_params(top='off', direction='out', width=1)
          plt.plot(wavelengths, ssa_halite_nk, label='ssa from nk and <D>' + ", <D> = " + str(
          plt.plot(wavelengths, wh, label='ssa gypsum')
          #plt.grid()
          plt.legend()
          plt.xlabel("Wavelength (nm)", fontsize = 16)
          plt.ylabel("SSA", fontsize = 16)
          plt.tick_params(labelsize = 12)
          plt.tight_layout()
          #plt.title("gypsum reference")
          #plt.savefig("./figs/fitt_ssa_gypsum.png", dpi=300)
```

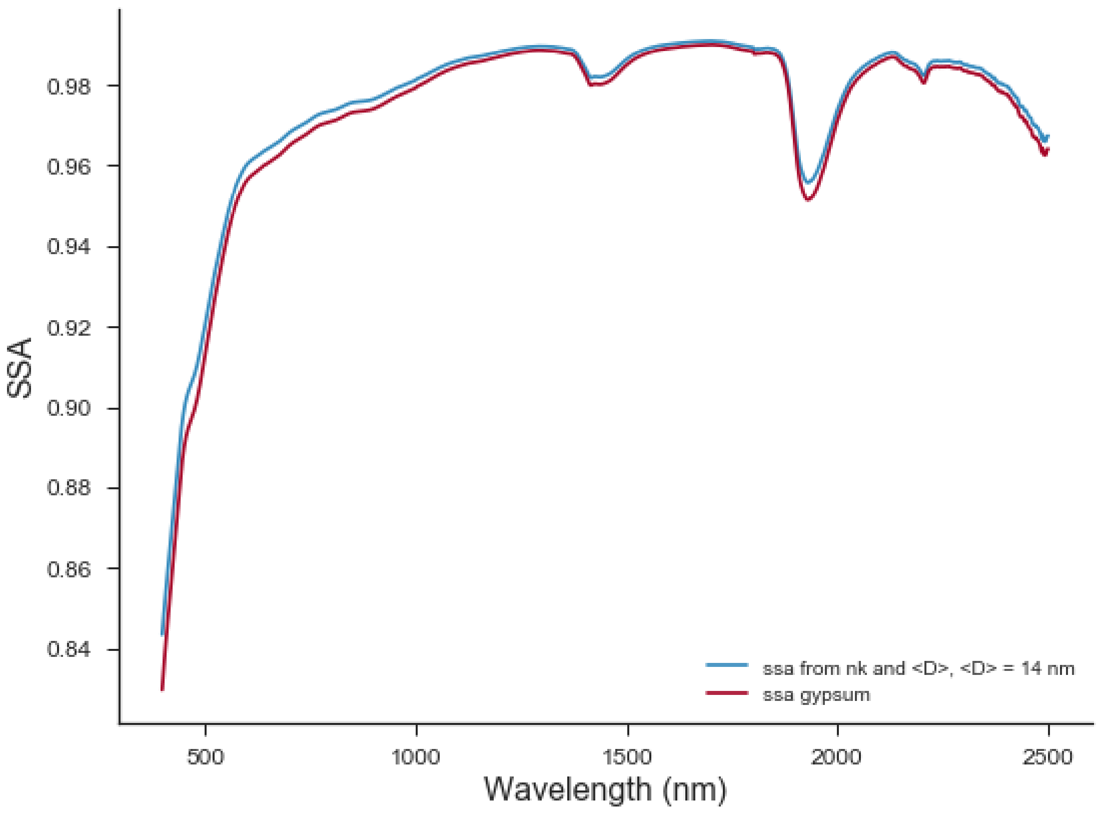

0.4.1 Find the mixing coefficients

0.4.2 Mixing Coefficients

$$\rho_{halite} = 2.16 gm/cc$$

$$\rho_{gypsum} = 2.31 gm/cc$$

$$\omega_{mix} = f_h \omega_h + f_g \omega_g$$

$$f_h = \frac{\sigma_h}{\sigma_h + \sigma_g}$$

$$f_g = \frac{\sigma_g}{\sigma_h + \sigma_g}$$

$$\sigma = \frac{m}{\rho D}$$

$$f_h = \frac{1}{1 + \frac{m_g \rho_h D_h}{m_h \rho_g D_g}}$$

$$f_m = \frac{m_g}{m_h}$$

$$f_d = \frac{D_g}{D_h}$$

$$f_h = \frac{1}{1 + \frac{f_m \rho_h}{\rho_g f_d}}$$

$$f_g = \frac{1}{1 + \frac{f_d \rho_g}{\rho_h f_m}}$$

```
In [257]: #wg075h025 = extract_ssa(r_g075h025)
          #wg05h05 = extract_ssa(r_g05h05)
          #wg025h075 = extract_ssa(r_g025h075)
          def residual_unmixing1(f):
              fm = f[0]
              dg = f[1]
              dh = f[2]
              fd = dg/dh
              ss = (1./(1.+fm*2.16/fd/2.31)*wi(h_n,h_k_min,dh, wavelengths) +
                    1./(1.+fd*2.31/2.16/fm)*wi(g_n,g_k_min,dg, wavelengths)-wg075h025)**2
              return ss.sum()
```

```
In [258]: ansunmix = minimize(residual_unmixing1, [0.5,20000,20000], method='nelder-mead', opt
          print(ansunmix.x)
```

```
Optimization terminated successfully.
         Current function value: 0.143907
         Iterations: 192
         Function evaluations: 393
[  2.33169332e+00   5.05112160e+04   7.32334513e+04]
```

```
In [259]: wg075h025_unmix = 1./(1.+ ansunmix.x[0]*2.16/(ansunmix.x[1]/ansunmix.x[2])/2.31)*wi(
```

```
In [260]: ssa_unimx1_fig = plotSpectrum([wavelengths,
                                          wavelengths],
                                         [wg075h025,
                                         wg075h025_unmix],
                                         ["ssa H0.25 G0.75",
                                         "ssa H0.25 G0.75 unmix" + " f$_m$ = " + str(ansunmix.x
                                         "Wavelength (nm)", "SSA",False,"./figs/ssa_data.png")
```

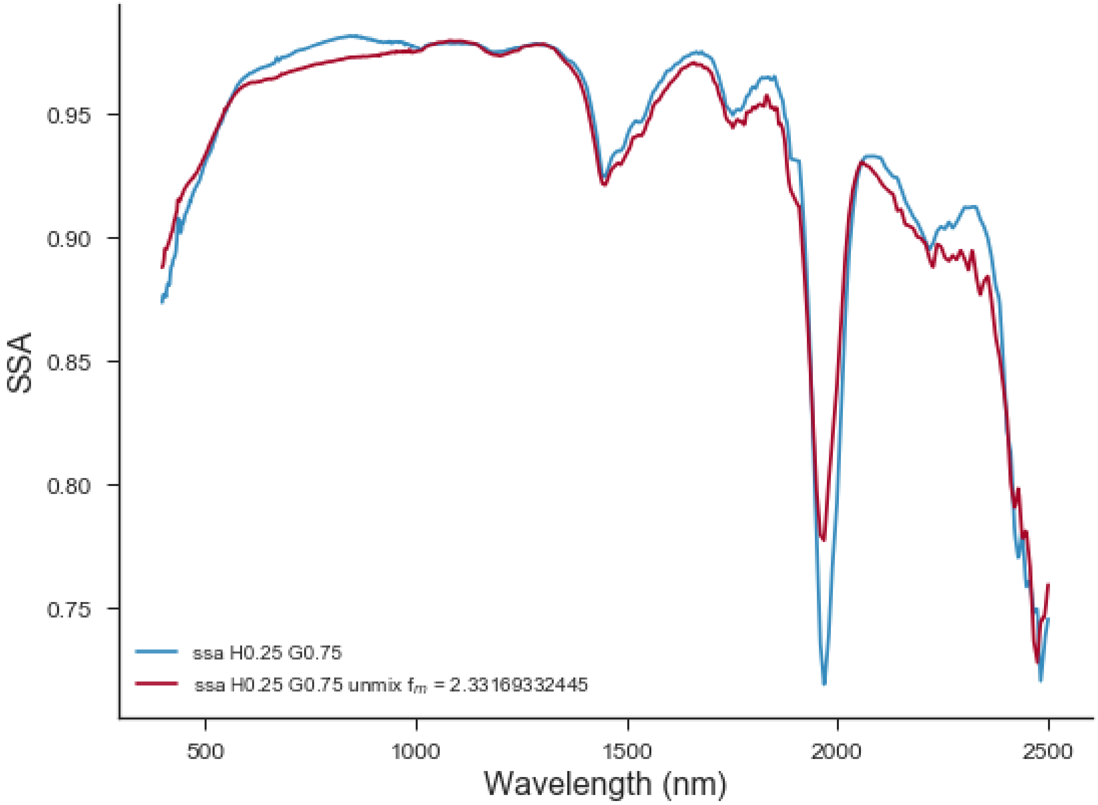


```
In [261]: mu0 = np.cos(incoming_angle*np.pi/180);
          mu = np.cos(emmerging_angle*np.pi/180);
          r_wg075h025_unmix = reflect(wg075h025_unmix,mu0,mu)

In [343]: ssa_unimx1_fig = plotSpectrum([wavelengths,
                                          wavelengths],
                                         [r_g075h025,
                                         r_wg075h025_unmix],
                                         ["r H0.25 G0.75",
                                         "r H0.25 G0.75 unmix" + " f$_m$ = " + str(ansunmix.x[0]
                                         "Wavelength (nm)", "Reflectance",True,"./figs/r_unmix1
```

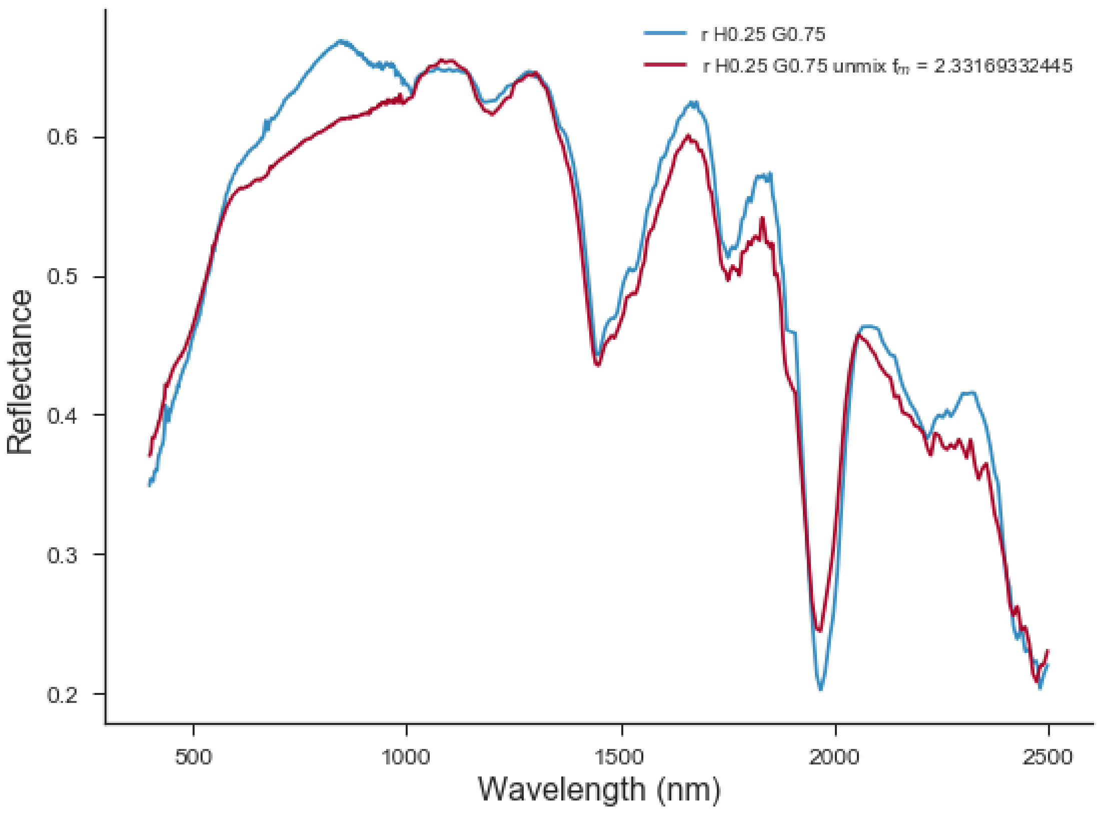


```
In [263]: bnds = ((0.5, None), (0, None),(0, None))
          def residual_unmixing1(f):
              fm = f[0]
              dg = f[1]
              dh = f[2]
              fd = dg/dh
              ss = (1./(1.+fm*2.16/fd/2.31)*wi(h_n,h_k_min,dh, wavelengths) +
                    1./(1.+fd*2.31/2.16/fm)*wi(g_n,g_k_min,dg, wavelengths)-wg075h025)**2
              return ss.sum()
```

```
In [269]: ansunmix2 = minimize(residual_unmixing2, [1,23000,23000], method='L-BFGS-B', options=
          print(ansunmix2.x)
```

```
[  7.20487619e-01   2.70226010e+05   1.18063460e+05]


C:\ProgramData\Anaconda3\lib\site-packages\ipykernel_launcher.py:8: RuntimeWarning: invalid va

C:\ProgramData\Anaconda3\lib\site-packages\ipykernel_launcher.py:10: RuntimeWarning: divide by
  # Remove the CWD from sys.path while we load stuff.
C:\ProgramData\Anaconda3\lib\site-packages\ipykernel_launcher.py:8: RuntimeWarning: divide by
```

```
In [270]: wg05h05_unmix = 1./(1.+ ansunmix2.x[0]*2.16/(ansunmix2.x[1]/ansunmix2.x[2])/2.31)*wi

In [271]: ssa_unimx2_fig = plotSpectrum([wavelengths,
                                         wavelengths],
                                        [wg05h05,
                                        wg05h05_unmix],
                                        ["ssa H0.5 G0.5",
                                        "ssa H0.5 G0.5 unmix" + " f$_m$ = " + str(ansunmix2.x[(
                                        "Wavelength (nm)", "SSA",False,"./figs/ssa_data.png")
```

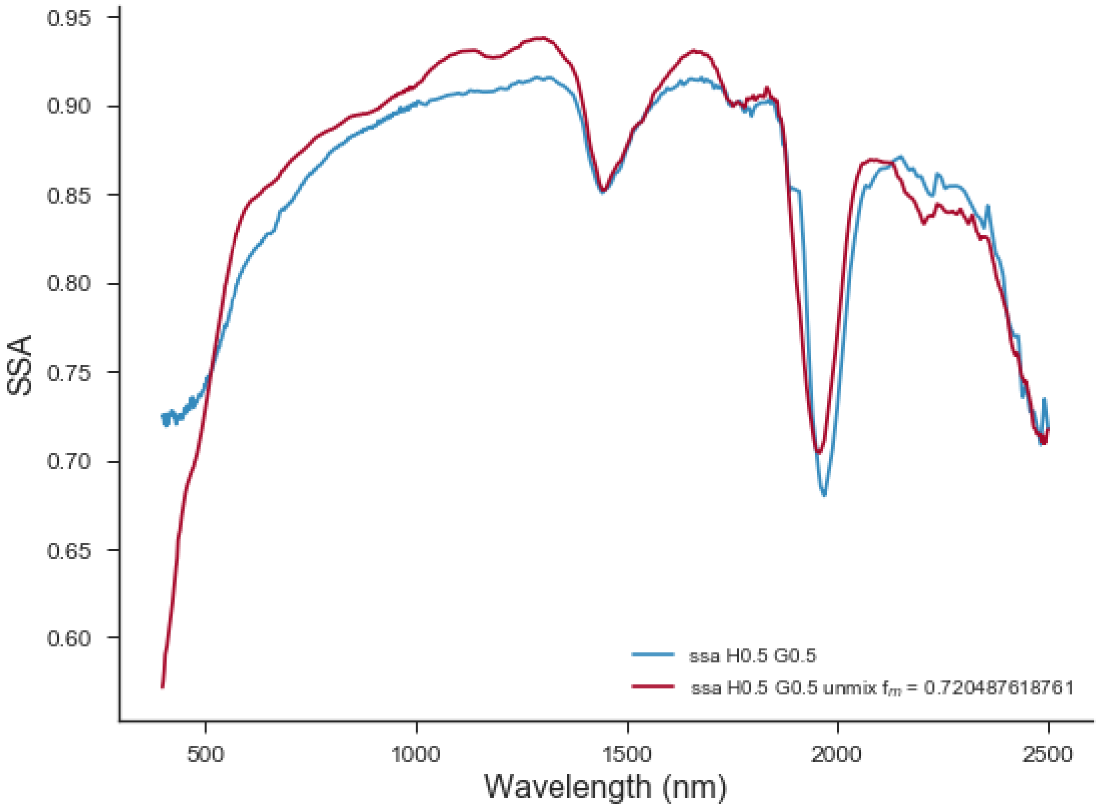


```
In [272]: r_wg05h05_unmix = reflect(wg05h05_unmix,mu0,mu)

In [344]: r_unimx2_fig = plotSpectrum([wavelengths,
                                       wavelengths],
                                      [r_g05h05,
                                      r_wg05h05_unmix],
                                      ["r H0.5 G0.5",
                                      "r H0.5 G0.5 unmix" + " f$_m$ = " + str(ansunmix2.x[0]]
                                      "Wavelength (nm)", "Reflectance",True,"./figs/r_unmix2
```

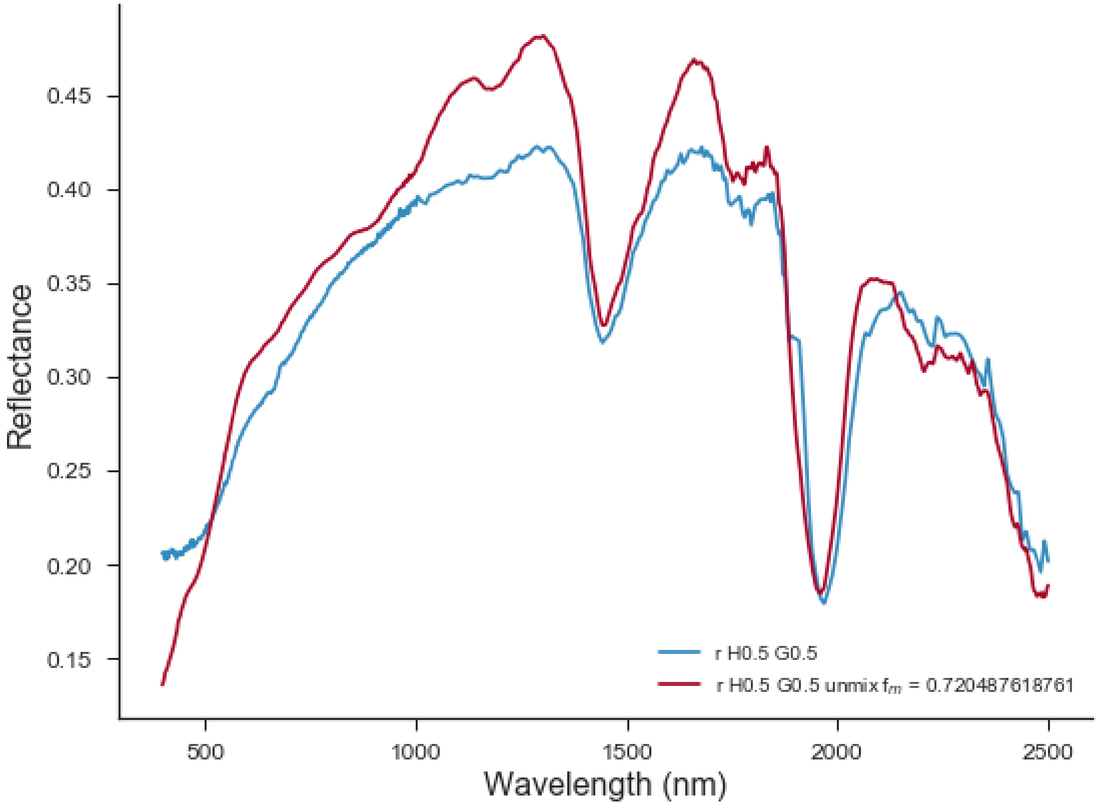


```
In [274]: bnds = ((0, None), (0, None),(0, None))
          def residual_unmixing1(f):
              fm = f[0]
              dg = f[1]
              dh = f[2]
              fd = dg/dh
              ss = (1./(1.+fm*2.16/fd/2.31)*wi(h_n,h_k_min,dh, wavelengths) +
                    1./(1.+fd*2.31/2.16/fm)*wi(g_n,g_k_min,dg, wavelengths)-wg025h075)**2
              return ss.sum()

In [366]: ansunmix3 = minimize(residual_unmixing3, [0.3,25000,29000], method='L-BFGS-B', optio
          print(ansunmix3.x)

C:\ProgramData\Anaconda3\lib\site-packages\ipykernel_launcher.py:1: OptimizeWarning: Unknown so
  """Entry point for launching an IPython kernel.
C:\ProgramData\Anaconda3\lib\site-packages\ipykernel_launcher.py:10: RuntimeWarning: divide by
  # Remove the CWD from sys.path while we load stuff.
C:\ProgramData\Anaconda3\lib\site-packages\ipykernel_launcher.py:8: RuntimeWarning: invalid val

C:\ProgramData\Anaconda3\lib\site-packages\ipykernel_launcher.py:8: RuntimeWarning: divide by
```

```
[  5.30348344e-01   1.60242882e+05   1.38375736e+05]
```

```
In [367]: wg025h075_unmix = 1./(1.+ ansunmix3.x[0]*2.16/(ansunmix3.x[1]/ansunmix3.x[2])/2.31)*w
```

```
In [368]: ssa_unimx3_fig = plotSpectrum([wavelengths,
                                          wavelengths],
                                         [wg025h075,
                                         wg025h075_unmix],
                                         ["ssa H0.75 G0.25",
                                         "ssa H0.75 G0.25 unmix" + " f$_m$ = " + str(ansunmix3.x
                                         "Wavelength (nm)", "SSA",True,"./figs/r_unmix3.png")
```

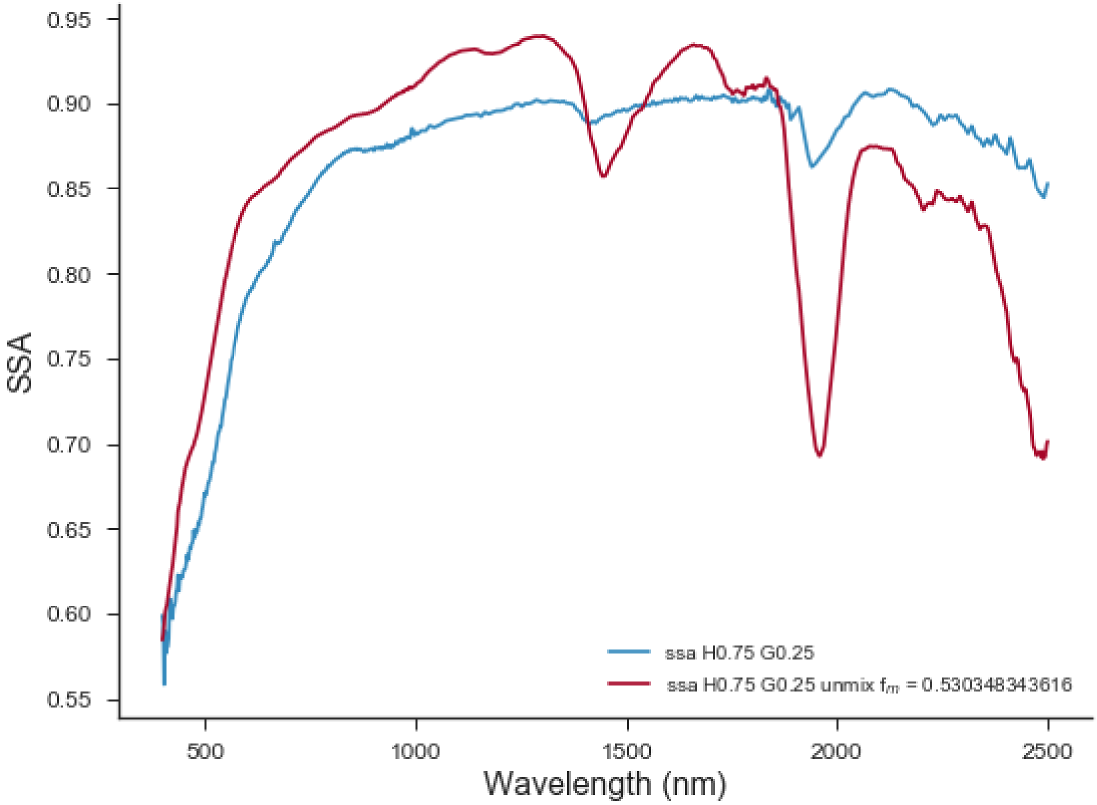


```
In [361]: r_wg025h075_unmix = reflect(wg025h075_unmix,mu0,mu)
```

```
In [362]: r_unimx3_fig = plotSpectrum([wavelengths,
                                        wavelengths],
                                       [r_g025h075,
                                       r_wg025h075_unmix],
                                       ["r H0.75 G0.25",
                                       "r H0.75 G0.25 unmix" + " f$_m$ = " + str(ansunmix3.x[(
                                       "Wavelength (nm)", "Reflectance",True,"./figs/r_unmix3
```

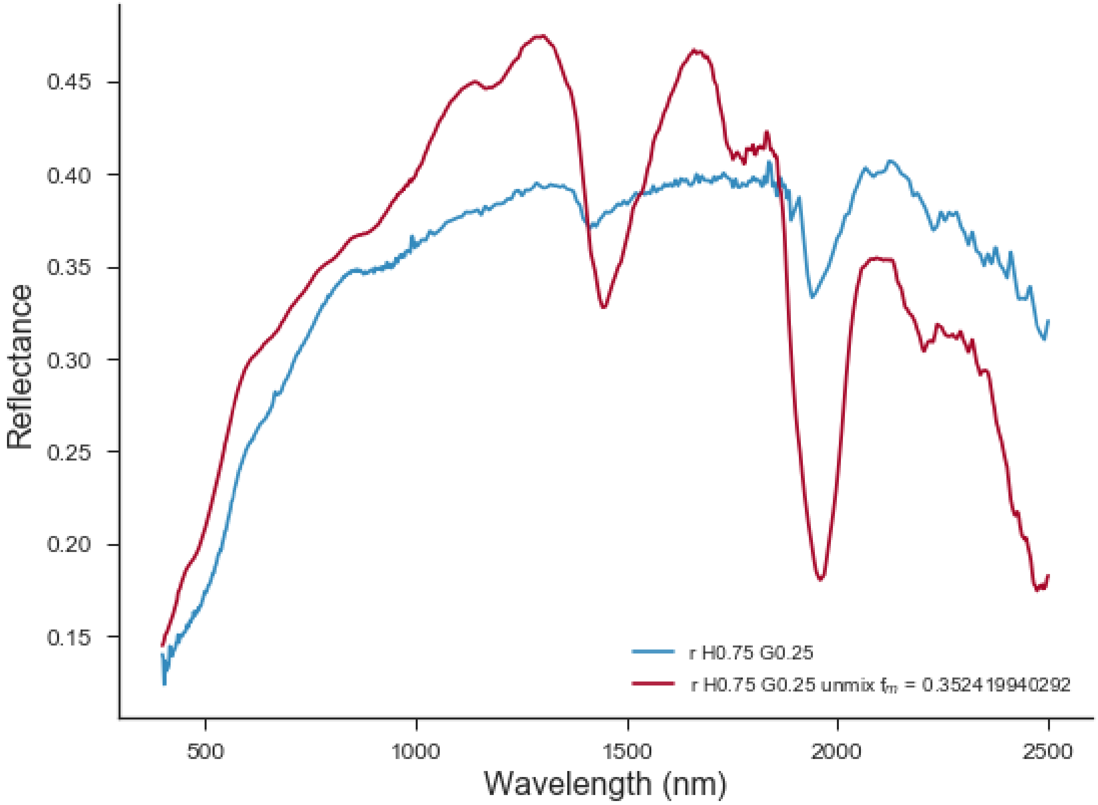
Reflectance
0.45
0.40
0.35
0.30
0.25
0.20
0.15
500
1000
1500
2000
2500
Wavelength (nm)
r H0.75 G0.25
r H0.75 G0.25 unmix f_m = 0.352419940292
