# Supplementary figures and images for: Hapke-based computational method to enable unmixing of hyperspectral data of common salts
Source: Chem Cent J. 2018 Aug 9;12:90. doi: 10.1186/s13065-018-0460-z (PMC6085231; doi:10.1186/s13065-018-0460-z)

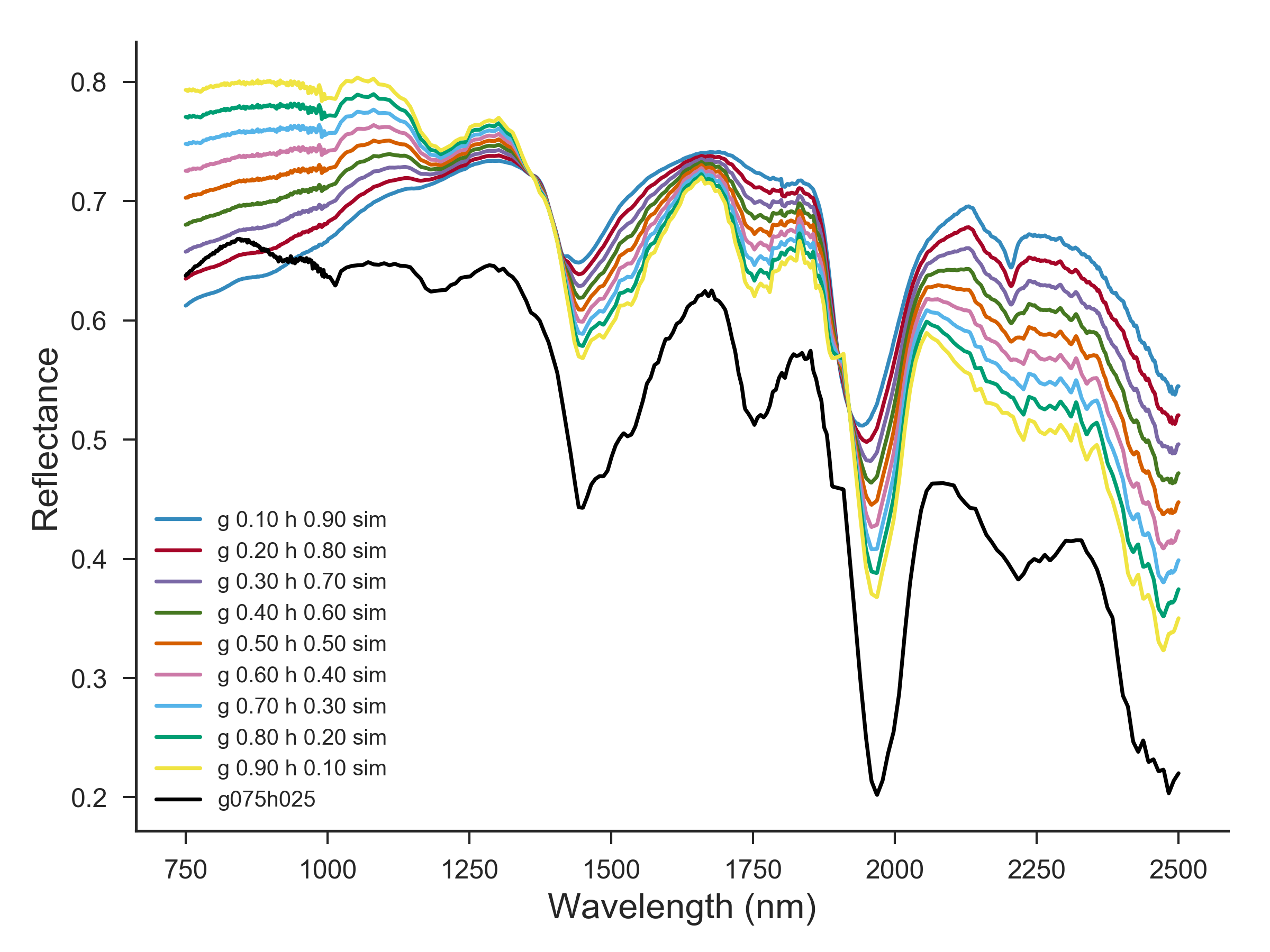

Supplement: Supplementary file 2 — Additional file 2. Additional details on the proposed fitting method and the used approach to simulate reflectance spectra. [file 13065_2018_460_MOESM2_ESM.png]
