# Supplementary material for: Hapke-based computational method to enable unmixing of hyperspectral data of common salts
Source: Chem Cent J. 2018 Aug 9;12:90. doi: 10.1186/s13065-018-0460-z (PMC6085231; doi:10.1186/s13065-018-0460-z)

## 1. Baseline removal and band estimation

###
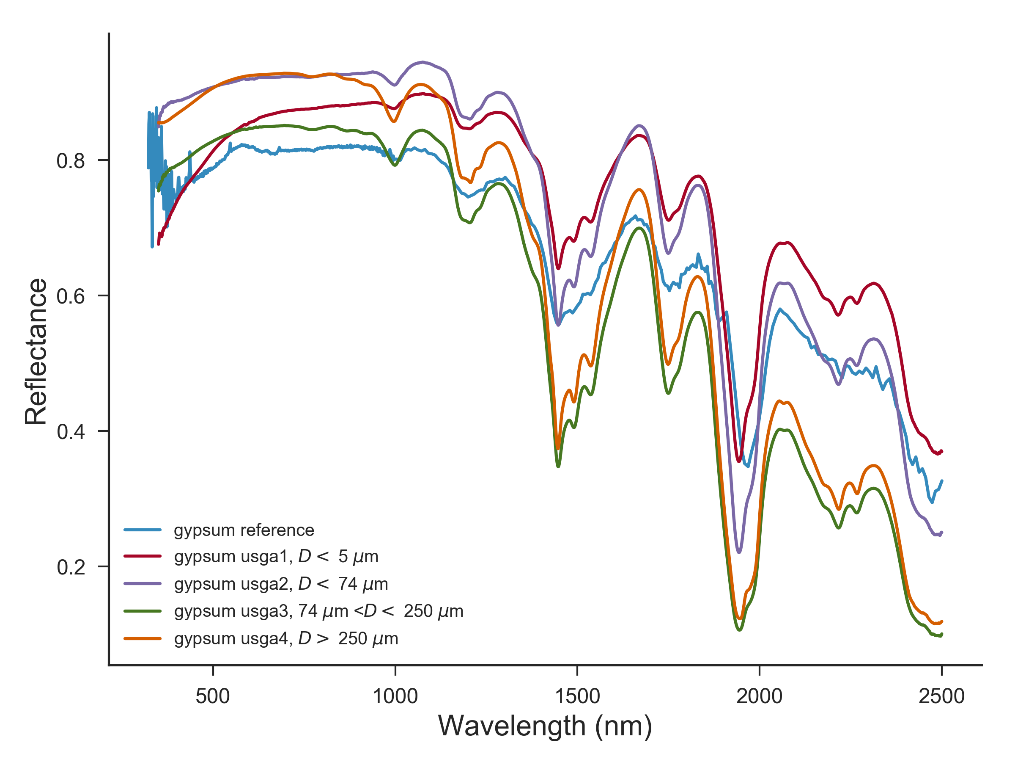
1.1 Endmember spectra


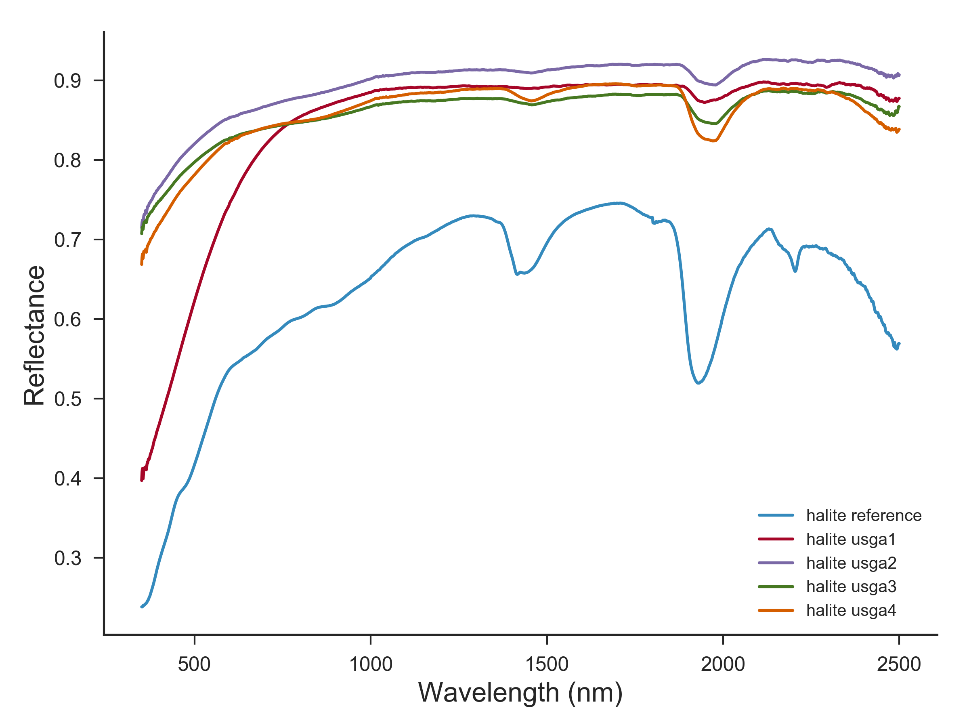


Gypsum has multiple absorption bands in the 300-2500nm wavelength range, making it easily identifiable (Hunt 1971, Clark 1999). The spectral frequencies are associated with the vibrational modes of the water molecules in the mineral structure. Similarly, the molecular vibration of water (O-H bonds vibrations) leads to absorption dips in the reflectance spectra of halite. However, there are noted differences between the two spectra making the distinction between the two minerals possible. To determine the band position
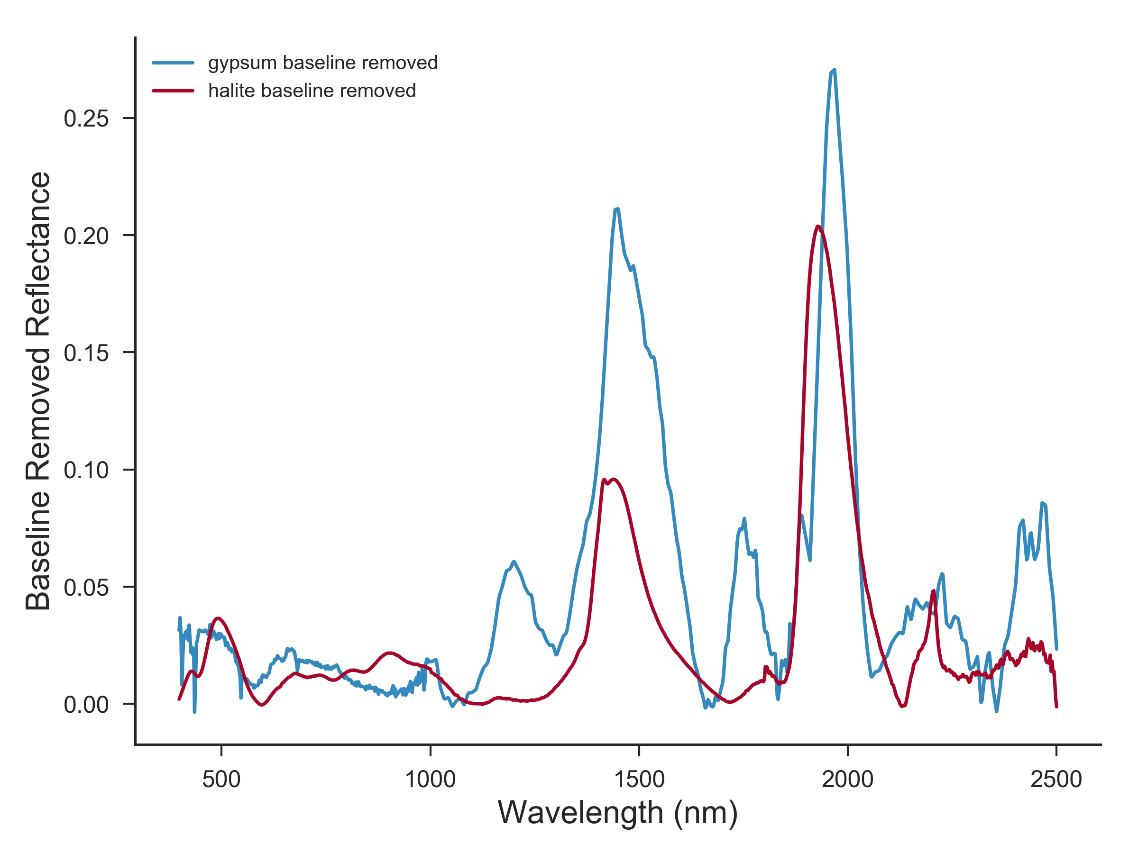
we extracted the continuum spectra by iteratively polynomial fitting of the reflectance data. This procedure helps in removing the shifts in the position of the bands due to the different slopes of continuum baseline spectra of the minerals.


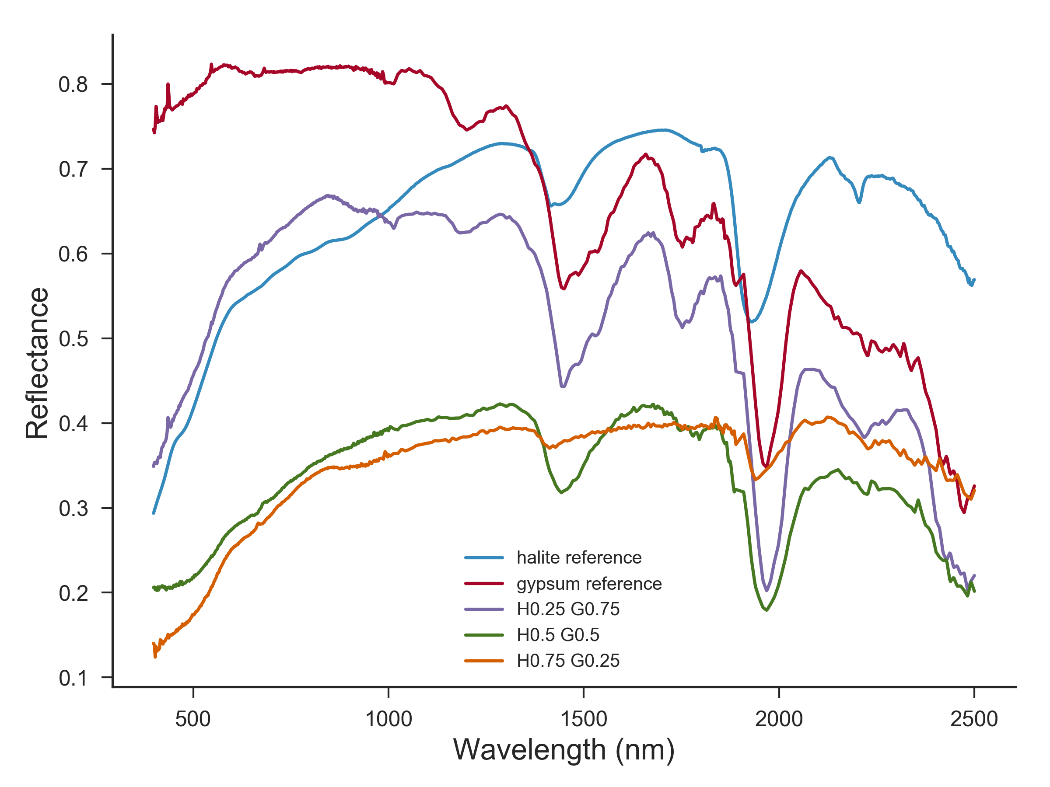
With the baseline removed, the spectra shows that Halite and Gypsum have common bands at 1450 nm, 1950 nm and 2200nm. However, for the gypsum the 1450, 1950 and 2200 bands consist of up to tree overlapped bands. In addition, gypsum has distinct absorption bands at 950nm, 1200nm and 1750nm.


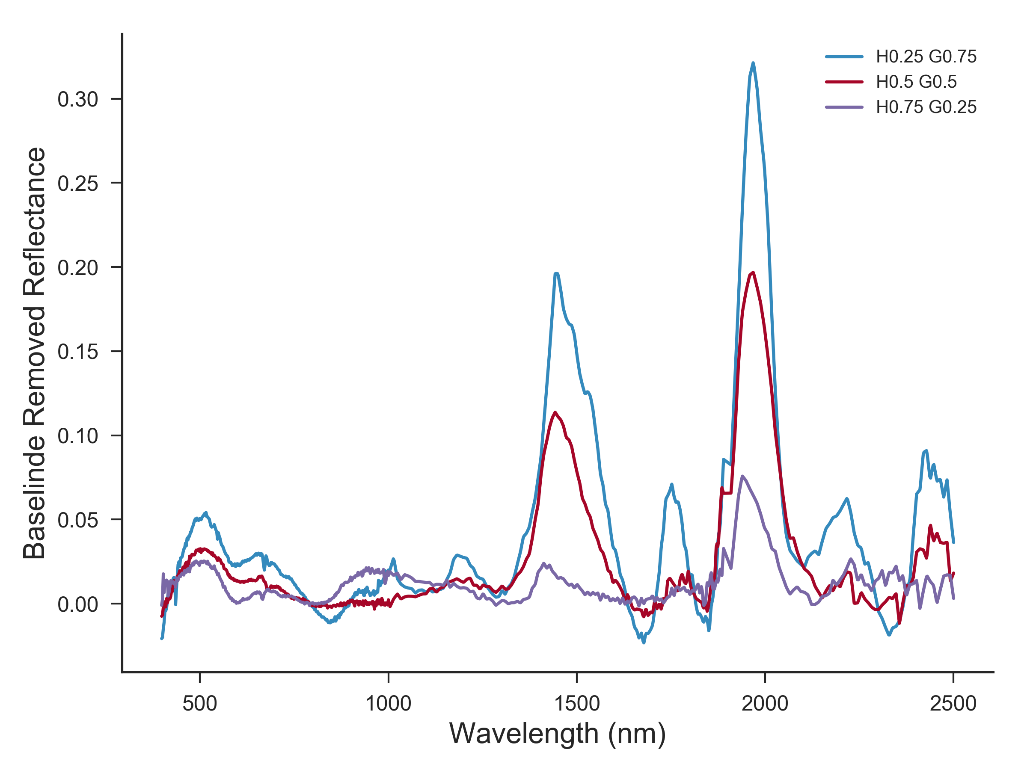
The thee distinct peaks make the detection of the gypsum presence in the mixtures recognizable. With the band depths depending on the gypsum concentration in the mixture.

The absorption bands and the overall spectral shape of the reflectance are not depending only on the material composition but also on the material physical properties (grain size, shape, etc)

To “unmix” the VIS-NIR reflectance spectra we use the Hapke radiative transfer model. In the simplest model which assume isotropic scatterers and no backscattering, the reflectance depends on the detection geometry and a single parameter, the single-scattering albedo which describe the reflectivity of a single grain.

$$r\left( \mu, \mu_{0} \right)=\frac{\omega}{4\left( \mu+\mu_{0} \right)}H\left( \mu\right)H(\mu_{0})$$

$$H\left( x \right)= \frac{1}{1-\omega x\left[ r_{0}+\frac{1-2r_{0}x}{2}\ln\left( \frac{1+x}{x} \right) \right]}$$

$$r_{0}=\frac{1-\sqrt{1-\omega}}{1+\sqrt{1-\omega}}$$

- $r$reflectance
- $\omega$ single-scattering albedo, $(0\leq\omega\left( \lambda\right)\leq1)$ fraction of the scattered light to incident light by a single particle
- $\mu_{0}$ cosine of the incident angle
- $\mu$ cosine of the emergence angle
- $H$ Chandrasekhar integral function, multiple scattering function

In turn, the single-scattering albedo will depend on the effective grain size (D) and the optical constants (n, k) on the material

$$\omega=S_{e}+\left( 1-S_{e} \right)\frac{1-S_{i}}{1-S_{i}\Theta}\Theta$$

Where $S_{e}$ is the surface reflection coefficient for externally incident light

$$S_{e}=\frac{\left( n-1 \right)^{2}+k^{2}}{\left( n+1 \right)^{2}+k^{2}}$$

$S_{i}$ the reflection coefficient for internally scattered light:

$$S_{i}=1-\frac{4}{n\left( n+1 \right)^{2}}$$

$\Theta$ the transmission function of the grain:

$$\Theta=e^{-i\alpha D}$$

$$\alpha=\frac{4k\pi}{\lambda}$$

with $n$ the real part of the material complex optical index, k the imaginary part of the material complex optical index, $\lambda$the wavelength of the photons, D the diameter of the grain.

Now, the single-scattering albedo of a mixture of grains $\omega_{mix}$, is a linear combination of the single-scattering albedos of its individual endmembers,$\omega_{i}$:

$$\omega_{mix} =\sum_{i} f_{i}\omega_{i}$$

fractional relative cross section of component i:

$$f_{i}=\frac{\sigma_{i}}{\sum_{i} \sigma_{i}}$$

$$\sigma_{i}=\frac{m_{i}}{\rho_{i}D_{i}}$$

$m_{i}$ - mass abundance,

$\rho_{i}$ - density,

$D_{i}$ - grain size

Thus, the reflectance spectra can be inverted to determine the mass abundance and grain sizes of the endmembers in the mixture.


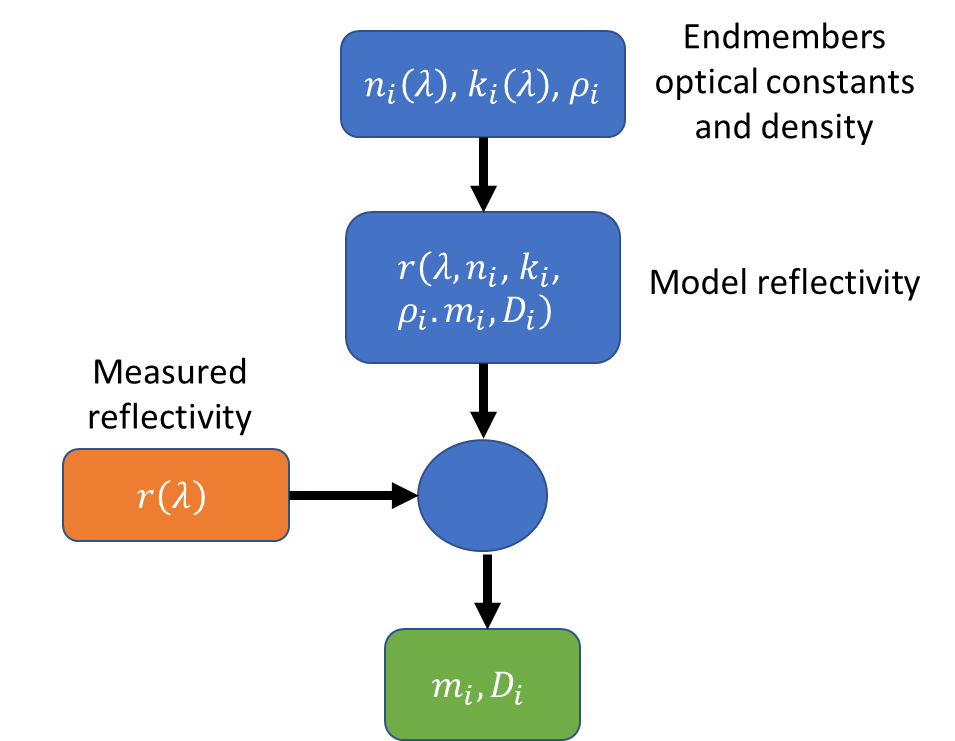
Knowledge of the endmembers optical constants and density is required to fit the reflectance model to the data and extract mass abundances and grain sizes.


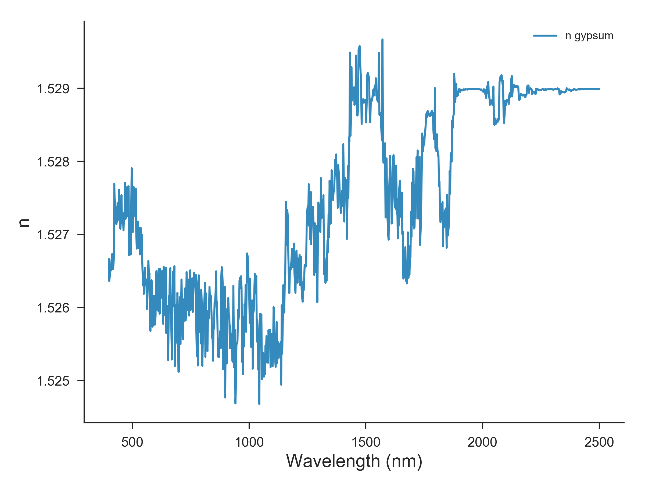

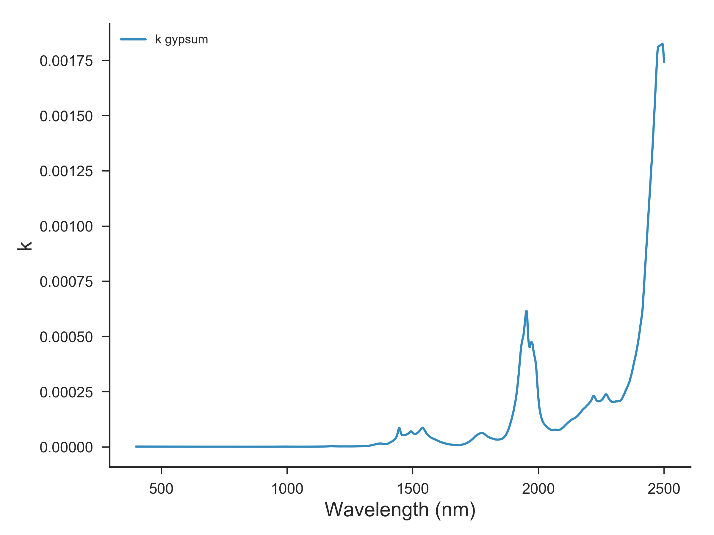
Example gypsum (n, k) data from Roush (2005):


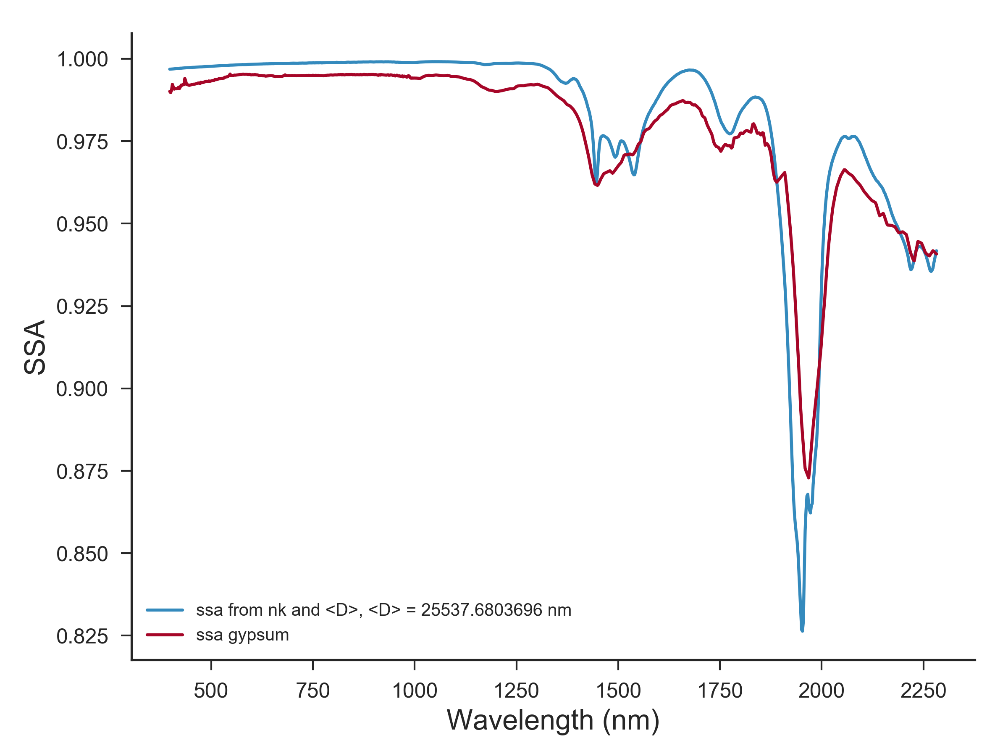
Using n, and k and ssa determined from reflectance data determined the grain effective diameter D:


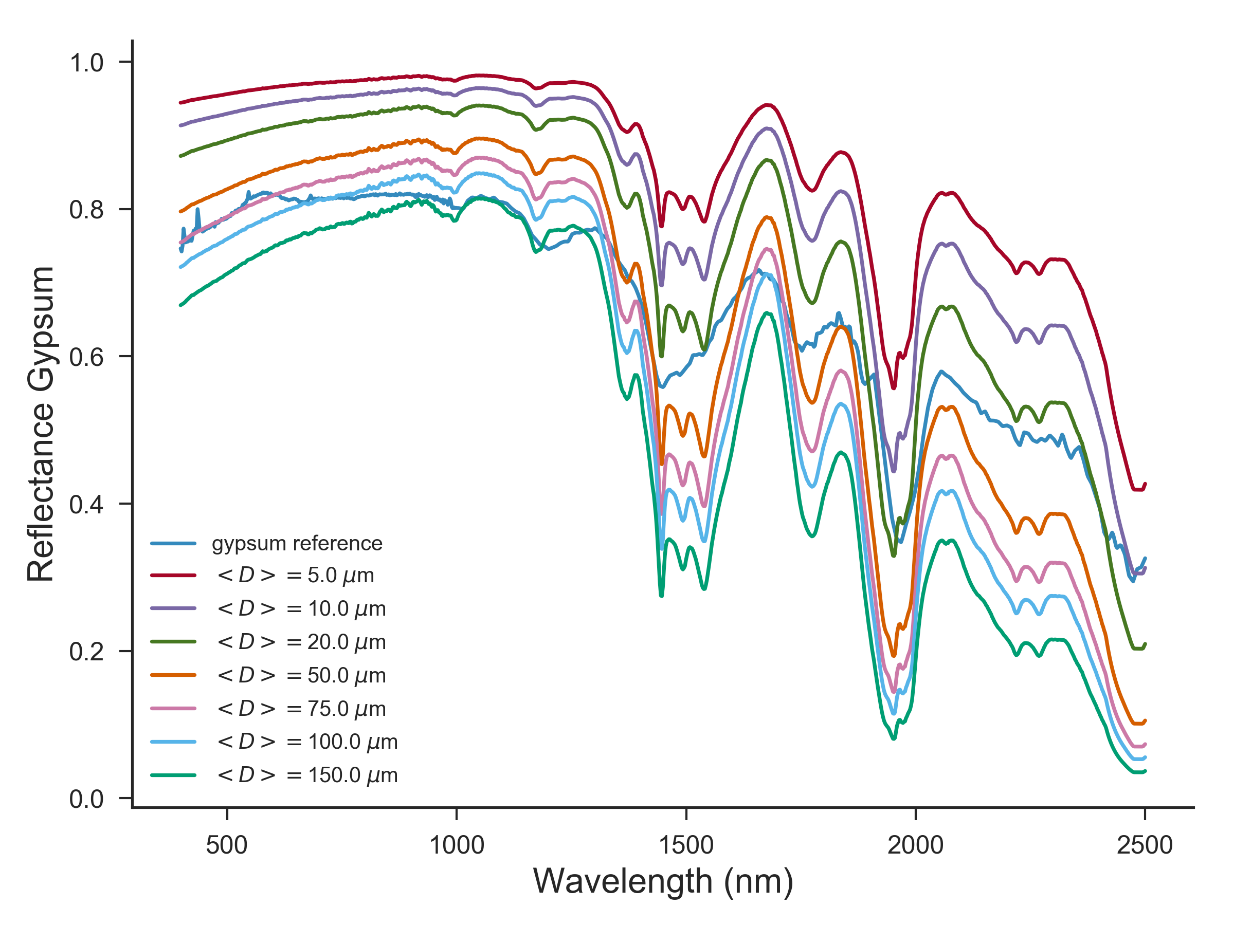
Calculated gypsum reflectance for different grain sizes and the gypsum data:


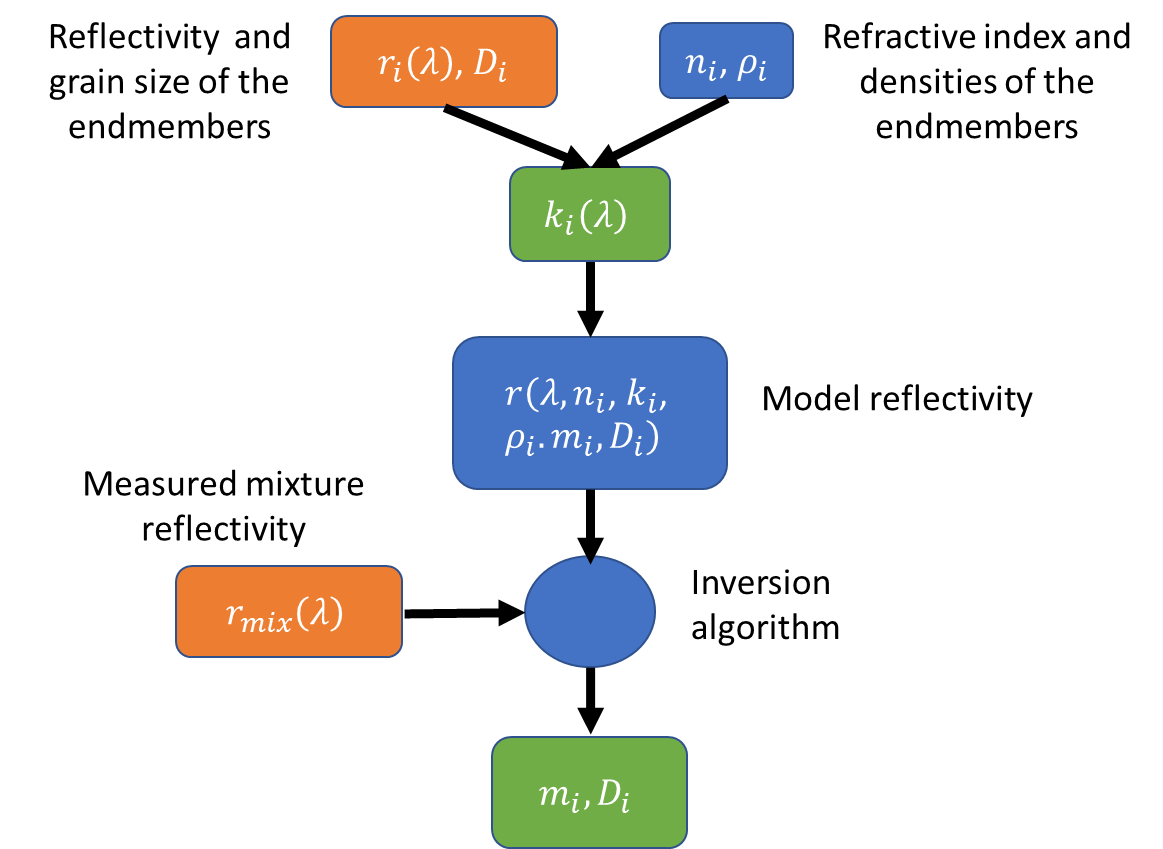
Alternatively, for some materials the extinction coefficient is not known, while the density and the real part of the refractive index can be found in material databases. In the first approximation the refractive index can be assumed constant as it varies slowly in the VIS-NIR range. Using the measured reflectanceses of endmembers materials with known grains sizes the single-scattering model functions can be inverted to determine the extinctions coefficients. Now, we have all parameters required to fit the reflectance of the mixture materials.

## Inversion Algorithms

There are two general algorithms for extracting the mass abundances and the grain sizes of the endmembers in the mixture from the model and measured reflectance.

One is an optimization algorithm that searches the fitting parameters $m_{i},D_{i}$ that would minimize the root mean square of the difference between the model and data reflectanceses.

Second method is the probabilistic method, that uses Markov Chain Monte Carlo algorithm and Bayes Theorem to estimate the probability density functions of the model parameters given the reflectance data and model relationship between parameters. One of the advantages of the probabilistic model is that the detection noise model (which can be non-gaussian for low count photons per pixel) can be accounted in the calculations.

While the fist approach supplies a single set of data for the endmembers mass fractions and particles sizes the probabilistic model gives a range of values and in principle can account for non-unique solutions in the model parameters.

### Optimization Method

We write the mixing ssa

$$\omega_{mix}=c\omega_{halite}+\left( 1-c \right)\omega_{gypsum}$$

Try to reduce the dependence on the grain size to a shift in the reflectance spectra. (not quite valid assumption). Determine the mixing fraction c from the scaled reflectances minimizing:

$$\sqrt{{\sum_{\lambda_{i}} \left( \frac{r_{mix}\left( \lambda_{i} \right)}{r_{mix}^{max}}-\frac{r_{data}\left( \lambda_{i} \right)}{r_{data}^{max}} \right)}^{2}}$$

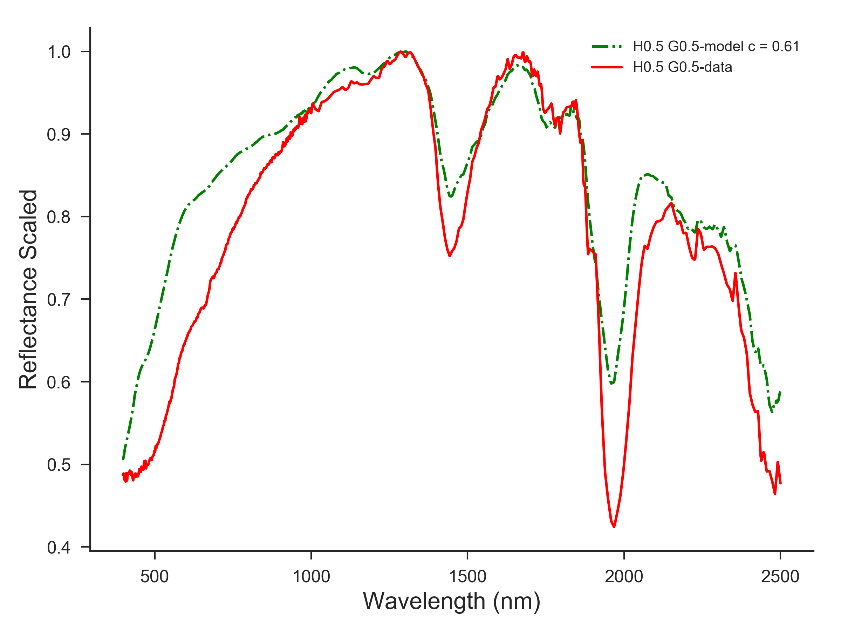


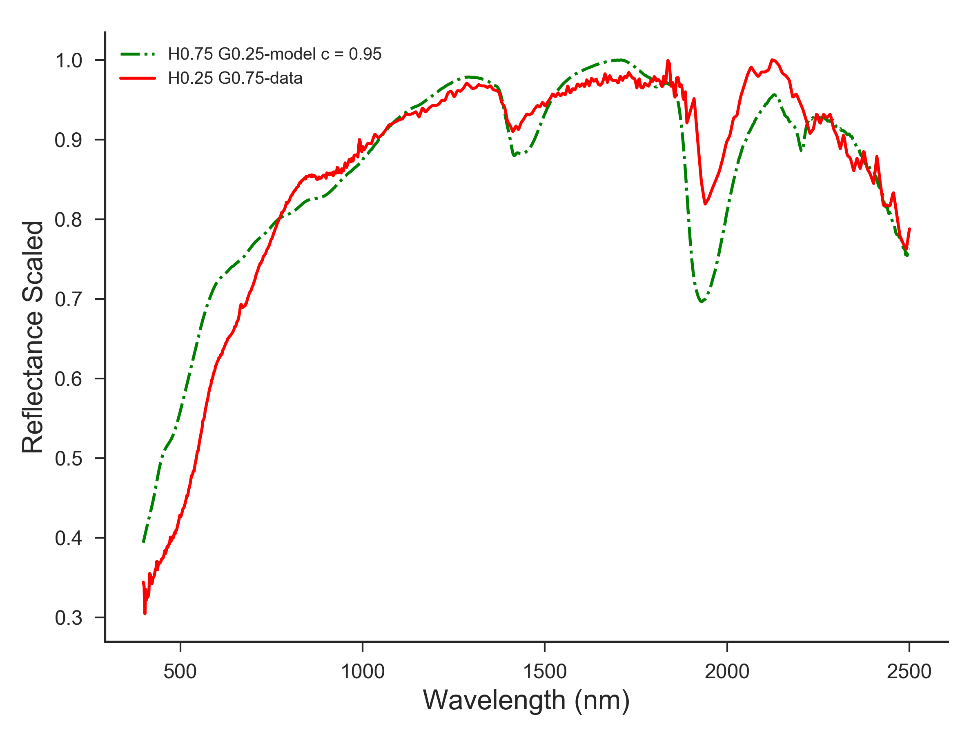


## Bayesian approach


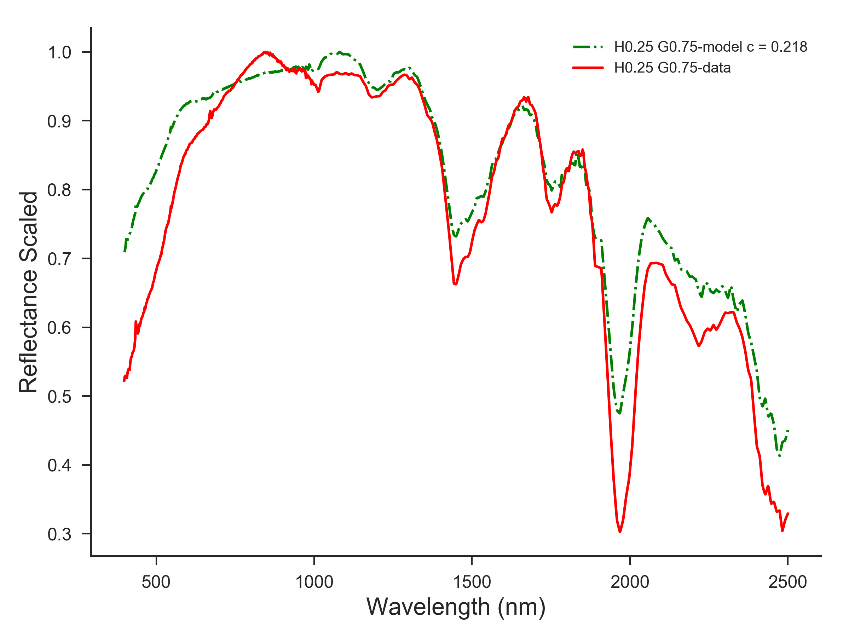
Assume that the ssa is given by the Hapke model plus a random noise $\epsilon$:

$$\omega\left( \lambda_{i} \right)=c_{halite}\omega_{halite}+c_{gypsum}\omega_{gypsum}+\epsilon$$

The likelihood function for the observations $\omega_{data}\left( \lambda_{i} \right)$ for the model assuming multivariate gaussian noise:

$$f\left( \omega_{data}|c_{h},c_{g} \right)=\frac{1}{\left( 2\pi\left| K \right| \right)^{2}}exp\left\{ -\frac{1}{2}\left( \omega_{data}-\omega_{model}\left( c_{h},c_{g} \right) \right)^{T}K^{-1}\left( \omega_{data}-\omega_{model}\left( c_{h},c_{g} \right) \right) \right\}$$

$c_{h},c_{g}$ are assumed to have uniform prior distribution $f(c_{h},c_{g})$. And use Bayes theorem to determine the posterior distribution:

$$f\left( c_{h},c_{g}|\omega_{data} \right)=\frac{f\left( \omega_{data}|c_{h},c_{g} \right)f(c_{h},c_{g})}{f(\omega_{data})}$$

The factors for H0.5G05 data:


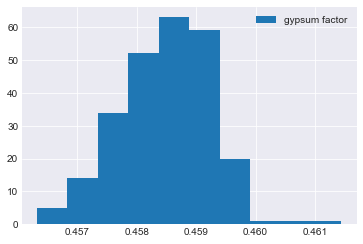


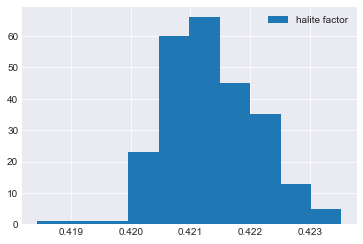

Supplement: Supplementary file 3 — Additional file 3. Simulation of reflection spectra for non-intimate linear mixture for comparison. [file 13065_2018_460_MOESM3_ESM.docx]
